# Polymeric viologen-based electron transfer mediator for improving the photoelectrochemical water splitting on Sb_2_Se_3_ photocathode

**DOI:** 10.1016/j.fmre.2022.03.013

**Published:** 2022-04-01

**Authors:** Chang Liu, Fusheng Li, Linqin Wang, Zeju Li, Yilong Zhao, Yingzheng Li, Wenlong Li, Ziqi Zhao, Ke Fan, Fei Li, Licheng Sun

**Affiliations:** a State Key Laboratory of Fine Chemicals, Institute of Artificial Photosynthesis, DUT-KTH Joint Education and Research Centre on Molecular Devices, Institute for Energy Science and Technology, Dalian University of Technology, Dalian 116024, China; b Center of Artificial Photosynthesis for Solar Fuels, School of Science, Westlake University, Hangzhou 310024, China; c Department of Chemistry, School of Engineering Sciences in Chemistry, Biotechnology and Health, KTH Royal Institute of Technology, Stockholm 10044, Sweden

**Keywords:** Viologen, Electron transfer mediator, Sb_2_Se_3_ photocathode, Solar water splitting, Hydrogen evolution reaction

## Abstract

The photogenerated charge carrier separation and transportation of inside photocathodes can greatly influence the performance of photoelectrochemical (PEC) H_2_ production devices. Coupling TiO_2_ with p-type semiconductors to construct heterojunction structures is one of the most widely used strategies to facilitate charge separation and transportation. However, the band position of TiO_2_ could not perfectly match with all p-type semiconductors. Here, taking antimony selenide (Sb_2_Se_3_) as an example, a rational strategy was developed by introducing a viologen electron transfer mediator (ETM) containing polymeric film (poly-1,1′-dially-[4,4′-bipyridine]-1,1′-diium, denoted as PV^2+^) at the interface between Sb_2_Se_3_ and TiO_2_ to regulate the energy band alignment, which could inhibit the recombination of photogenerated charge carriers of interfaces. With Pt as a catalyst, the constructed Sb_2_Se_3_/PV^2+^/TiO_2_/Pt photocathode showed a superior PEC hydrogen generation activity with a photocurrent density of −18.6 mA cm^−2^ vs. a reversible hydrogen electrode (RHE) and a half-cell solar-to-hydrogen efficiency (HC-STH) of 1.54% at 0.17 V vs. RHE, which was much better than that of the related Sb_2_Se_3_/TiO_2_/Pt photocathode without PV^2+^ (−9.8 mA cm^−2^, 0.51% at 0.10 V vs. RHE).

## Graphical abstract

PV^2+^ electron-transfer mediator (ETM) layer introduced into Sb_2_Se_3_/TiO_2_ interface, leading to a superior PEC hydrogen generation activity through tuning the Sb_2_Se_3_/TiO_2_ energy band alignment and inhibiting recombination of charge carriers efficiently.

**Figure:**
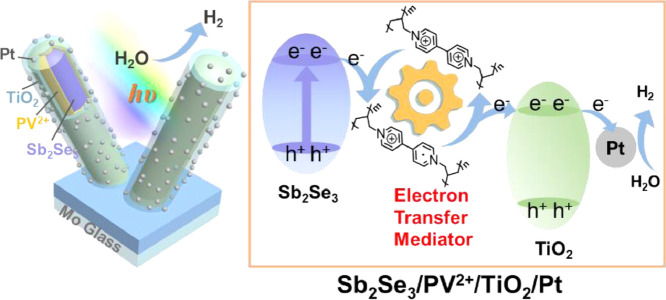

## 1 Introduction

The storage of solar energy into chemical bonds via artificial photosynthesis represents one of the most promising approaches for realizing a sustainable energy society [1,2]. The hydrogen (H_2_) production by water splitting is an attractive approach because of its carbon-neutral reaction feature and high availability of water. Solar H_2_ production can be achieved using a photoelectrochemical (PEC) device, where the functions of a light harvester and an electrolyzer can be integrated into a single device. Light-harvesting materials such as semiconductors can be used in PEC devices to absorb photons and produce electron-hole pairs, which drive the electrochemical reaction at the interface of photoelectrode–electrolyte. The efficiency of a PEC device can be comprehensively determined by its light-harvesting efficiency, charge separation efficiency, charge transfer rate, and surface reaction kinetics [3]. Up to now, numerous p-type semiconductors have been developed as photocathode materials for PEC H_2_ production, such as Si [4], [5], metal oxides [6], [7], Cu-based chalcogenides [8], [9], and polymers [10], [11], [12]. To improve the PEC performance, an n-type semiconductor is commonly decorated onto the p-type semiconductor to suppress charge recombination and promote charge separation by forming a p-n junction [13]. Particularly, TiO_2_ is a superior n-type semiconductor (bandgap ≈ 3.2 eV) with a wide pH window for water splitting, and an outstanding visible-light transmittance, making it broadly decorated onto various p-type semiconductors as a protective layer and an n-type junction layer [14,15]. However, the scarcity-related issues make it hard to choose a suitable n-type semiconductor that perfectly matches the band positions of a given p-type semiconductor.

For instance, chalcogenide antimony selenide (Sb_2_Se_3_) possesses all the requisites as a promising light-absorbing material for PEC water splitting applications. In detail, it has abundant and low-toxicity constituents, exhibits a bandgap (ca. 1.2 eV, covering the solar spectrum up to 1200 nm) with a desirable absorption co-efficiency (> 10^5^ cm^−1^ at ∼600 nm), which is capable of full light absorption within a thin layer, and demonstrates excellent hole mobility (10 cm^2^ V^−1^s^−1^ along the *c*-axis direction, enabling efficient charge transport) [16]. To date, efficient Sb_2_Se_3_-based photocathodes for PEC H_2_ production have been reported, and TiO_2_ was commonly used to improve the stability of Sb_2_Se_3_-based photocathodes [17]. However, because of the large conduction band (CB) offsets of the Sb_2_Se_3_/TiO_2_ junction [18], [19], the energy band alignment of the Sb_2_Se_3_/TiO_2_ interface forms a large cliff-like barrier for the electron transport process, which limits the PEC performance of Sb_2_Se_3_/TiO_2_-based photocathodes [20], [21]. Thus, additional buffer layers have generally been inserted between Sb_2_Se_3_ and TiO_2_ to adjust the band-alignment, thereby promoting carrier transport [22], [23]. However, an ideal buffer material that perfectly matches the band positions of Sb_2_Se_3_ and TiO_2_ is not easy to find. At present, only a few semiconductors, such as CdS, In_2_S_3_ and fullerene, have been reported [22], [23], [24]. Therefore, it is worth developing feasible methods to tune the energy band alignment of the p-n heterojunctions such as Sb_2_Se_3_/TiO_2_.

In this regard, viologen derivatives (1,1′-disubstitutes-[4,4′-bipyridine]-diium, V^2+^) are distinctive electron-transfer mediators (ETMs) that have attracted tremendous research interest, due to their excellent electrochemical properties [25]. When a single V^2+^ ion accepts an electron, correspondingly, a stable cationic radical (V^+•^) can be formed. This radical can be easily re-oxidized to V^2+^ after donating one electron to others [25], [26]. The long-lived redox-separated state of V^2+^/V^+•^ affords an outstanding electron-transfer capability; and it can promote charge separation of light absorption materials [27]. Therefore, it is very feasible to apply viologen derivative-based ETMs for overcoming the large electron transport barrier across the Sb_2_Se_3_/TiO_2_ interface, which could help to expand the scope of buffer layer materials.

**Scheme 1:**
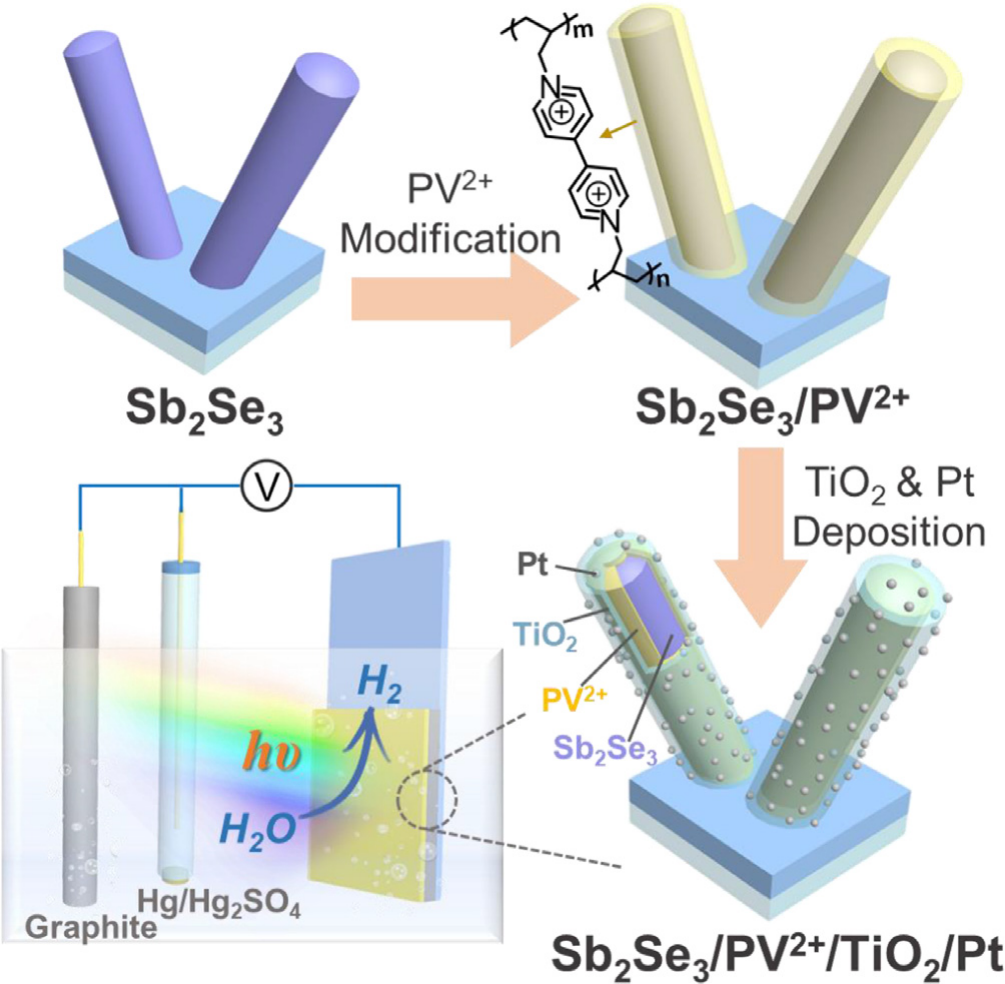
**Schematic diagram of the preparation procedure for Sb**_**2**_**Se**_**3**_**/PV**^**2+**^**/TiO**_**2**_**/Pt photocathode and related PEC cell.**

Herein, photocathodes based on the Sb_2_Se_3_/TiO_2_ heterojunction with Pt nanoparticle as catalyst were investigated for PEC H_2_ production. As shown in Scheme 1, a viologen-containing polymer layer (poly-1,1′-dially-[4,4′-bipyridine]-1,1′-diium, denoted as PV^2+^) was introduced between the interface of Sb_2_Se_3_ and TiO_2_. The resulting Sb_2_Se_3_/PV^2+^/TiO_2_/Pt photocathode exhibited a substantially improved PEC performance and a larger positive shift in the onset potential comparing to the related Sb_2_Se_3_/TiO_2_/Pt photocathode. The PV^2+^ ETM could effectively facilitate the charge separation and electron transfer in the Sb_2_Se_3_/TiO_2_ heterojunction. Our findings clearly suggest that polymeric viologen derivative-based ETMs have great potential as the buffer layer for the construction of high-efficiency Sb_2_Se_3_-based p-n heterojunction photocathodes toward water splitting. In addition, analogous ETM materials obtained by molecular engineering may be applicable for other heterojunction systems to promote charge separation and transportation.

## 2 Experiment

### 2.1 The preparation of Sb_2_Se_3_ film

Before Sb_2_Se_3_ thin film was deposited, the Mo glass was selenized using a homemade graphite box with 50 mg of Se powder under argon atmosphere at 490 °C for 1 min in a tube furnace. The Sb_2_Se_3_ absorber layer was grown on the selenized Mo-coated glass at 515 °C below 0.1 Pa by a close space sublimation (CSS) system. And then, the obtained Sb_2_Se_3_ film was treated in a selenourea solution (CH_4_N_2_Se, 5 mM) by a hydrothermal method at 95 °C for 2 h. The resulting film was taken out, rinsed with ethanol and water several times, and dried in the atmosphere.

### 2.2 The preparation of V^2+^

**Scheme 2:**
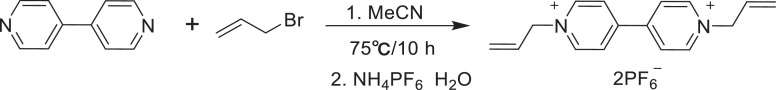
**Synthesis of V**^**2+**^**•2PF**^**6−**^**.**

The monomer of PV^2+^ was synthesized as shown in Scheme 2, in detail, 4,4′-bipyridine (500 mg), acetonitrile (MeCN, 50 mL) and excess allyl bromide (C_3_H_5_Br, ∼10 equivalent) were added into a flask. The mixture was refluxed at 100 °C for 10 h. After the reaction cooled to room temperature, the precipitate was collected, and washed by Et_2_O to remove the unreacted C_3_H_5_Br. Pure V^2+^•2Br^−^ could be obtained. Finally, V^2+^•2Br^−^ was dissolved in H_2_O (100 mL), and excess NH_4_PF_6_ solid was added. V^2+^•2PF^6−^ (named V^2+^) was precipitated and collected, which was washed by MeOH and Et_2_O several times. ^1^H NMR was shown in Fig. S9 (500 MHz, CD_3_CN): δ = 9.31 (*d, J* = 5 Hz, 4H), 8.75 (*d, J* = 10 Hz, 4H), 6.20 (*m*, 4H), 5.43 (*m*, 2H), 5.34 ppm (*d, J* = 5 Hz, 4H). ^13^C NMR was shown in Fig. S10 (125 MHz, CD_3_CN): δ = 123.49, 127.33, 129.91, 145.62, 150.29 ppm. FTIR (Fig. S11): 1639 cm^−1^ (C=N ring stretch vibration); 1651, 1562, 1509 and 1448 cm^−1^ (C=C stretch vibration); 1222 cm^−1^ (N−CH_2_ stretch vibration); 1002 cm^−1^ (CH_2_ wag vibration). HRMS (ESI): m/z calcd for C_16_H_18_F_6_N_2_P^+^: 383.1106; found: 383.1109 (Fig. S12).

### 2.3 The preparation of Sb_2_Se_3_/PV^2+^/TiO_2_/Pt and Sb_2_Se_3_/TiO_2_/Pt photocathodes

The Sb_2_Se_3_/PV^2+^ film was obtained by the photo-assisted electropolymerization method. Before photo-assisted electropolymerization, the deposition solution containing V^2+^•2PF^6−^ (0.25 mM) and NaClO_4_ (0.1 M) in MeCN was deaerated with argon for 15 min. The prepared Sb_2_Se_3_ film as working electrodes was cycled in successive scans from 0.2 to –1.4 V vs. Ag/AgCl under illumination (100 mW cm^−2^). The resulting electrode was rinsed with MeCN and ethanol, and dried by N_2_ flow. After that, a TiO_2_ layer (10 nm) was deposited on the top of the Sb_2_Se_3_/PV^2+^ film by an atomic layer deposition (ALD) system. The obtained film was denoted as Sb_2_Se_3_/PV^2+^/TiO_2_. The Sb_2_Se_3_/TiO_2_ sample was prepared by depositing the TiO_2_ layer on the surface of Sb_2_Se_3_ directly without the PV^2+^ layer.

Finally, Pt nanoparticles as catalysts were deposited onto the Sb_2_Se_3_/TiO_2_ and Sb_2_Se_3_/PV^2+^/TiO_2_ by the photo-assisted electrodeposition method. Briefly, the Sb_2_Se_3_/TiO_2_ and Sb_2_Se_3_/PV^2+^/TiO_2_ films were implemented in an H_2_PtCl_6_•6H_2_O solution (0.1 mM) with a constant current density of −8.5 μA cm^−2^ for 20 min under illumination. After deposition, the resulting electrodes were carefully washed with deionized water and dried by N_2_ flow. The corresponding electrodes were denoted as Sb_2_Se_3_/TiO_2_/Pt and Sb_2_Se_3_/PV^2+^/TiO_2_/Pt.

PEC measurements were performed in 0.1 M H_2_SO_4_ solution using a three-electrode configuration with a prepared electrode as the working electrode, a Hg/Hg_2_SO_4_ as the reference electrode, and graphite as the counter electrode. The potentials were converted to scales of reversible hydrogen electrode (RHE) according to $E_{\text{RHE}}\left(V\right)=E_{\left(\text{Hg}/H_{2}SO_{4}\right)}+E_{(\text{Hg}/H_{2}SO_{4}\text{vs}.\text{RHE})}$, $E_{\left(\text{Hg}/H_{2}SO_{4}\text{vs}.\text{RHE}\right)}$is the potential difference between Hg/Hg_2_SO_4_ electrode and a commercialized RHE (HydroFlex®). The calculated RHE was further estimated from the pH value of the electrolyte and confirmed according to the open-circuit voltage of a Pt net electrode (1 cm^2^) in the electrolyte with hydrogen bubbling. LSV curves of the samples were measured by an electrochemical workstation (CHI 760E) with a scan rate of 10 mV s^-1^ under the irradiation of a 100 W Xenon lamp (Newport Oriel LCS-100 solar simulator) equipped with an AM 1.5G filter. Applied bias photon-to-current efficiencies (ABPEs) of the photocathodes were calculated by using the LSV curves with an assumption of nearly 100% Faradaic efficiency, according to the equation of $\text{ABPE}(\%)=\frac{\left(0-E_{\text{applied}}\right)\times \left(J_{\text{photo}}-J_{\text{dark}}\right)}{P_{\text{input}}}\times 100\%$.

## 3 Results and discussion

The Sb_2_Se_3_ thin films were prepared via the closed-space sublimation (CSS) of Sb_2_Se_3_ powders onto a selenized Mo-coated glass. The obtained Sb_2_Se_3_ thin films were further treated with a solution of selenourea (CH_4_N_2_Se, 5 mM) at 95 °C for 2 h. The prepared Sb_2_Se_3_ thin film had a relatively smooth topography with a thickness of about 700 nm (Fig. S1). The X-ray diffraction (XRD) pattern of the obtained film displayed distinct diffraction peaks of Sb_2_Se_3_ (PDF: 15-0861) and the Mo substrate (Fig. S2). The orientations of the (211) and (221) planes exhibited strong diffraction patterns, corresponding to the highly [001]-textured *c*-axis crystal growth direction, indicating an excellent carrier-migration efficiency [28]. Furthermore, the Raman scattering spectrum (Fig. S3) exhibited the characteristic vibrations of orthorhombic Sb_2_Se_3_ with two main peaks centered at 188 and 208 cm^−1^, suggesting the Sb_2_Se_3_ has been obtained as expected [29]. The peak at 251 cm^−1^, meanwhile, indicated the existence of Sb_2_O_3_ or Se, which may have formed during measurement, and was commonly observed for Sb_2_Se_3_ films [30]. The surface chemical composition and bonding states of the as-prepared Sb_2_Se_3_ film were identified by X-ray photoelectron spectra (XPS) (Fig. S4). As shown in Figs. S5a and S5b, the prepared Sb_2_Se_3_ thin film exhibited similar Sb 3d and Se 3d spectra to Sb_2_Se_3_ films prepared by CSS and solution processes [31]. Scanning electron microscopy (SEM) images revealed that Sb_2_Se_3_ film had a nanorod-based structure (Fig. S6). Moreover, high-resolution transmission electron microscopy (HRTEM), as shown in Fig. S7, revealed that the interplanar d-spacing was 0.390 nm attributed to the (001) plane of orthorhombic Sb_2_Se_3_ [28]. Furthermore, transmission electron microscopy (TEM) images and corresponding energy-dispersive spectroscopy (EDS) analysis demonstrated that Sb and Se elements were distributed evenly throughout Sb_2_Se_3_ nanorod (Fig. S8).

**Fig. 1:**
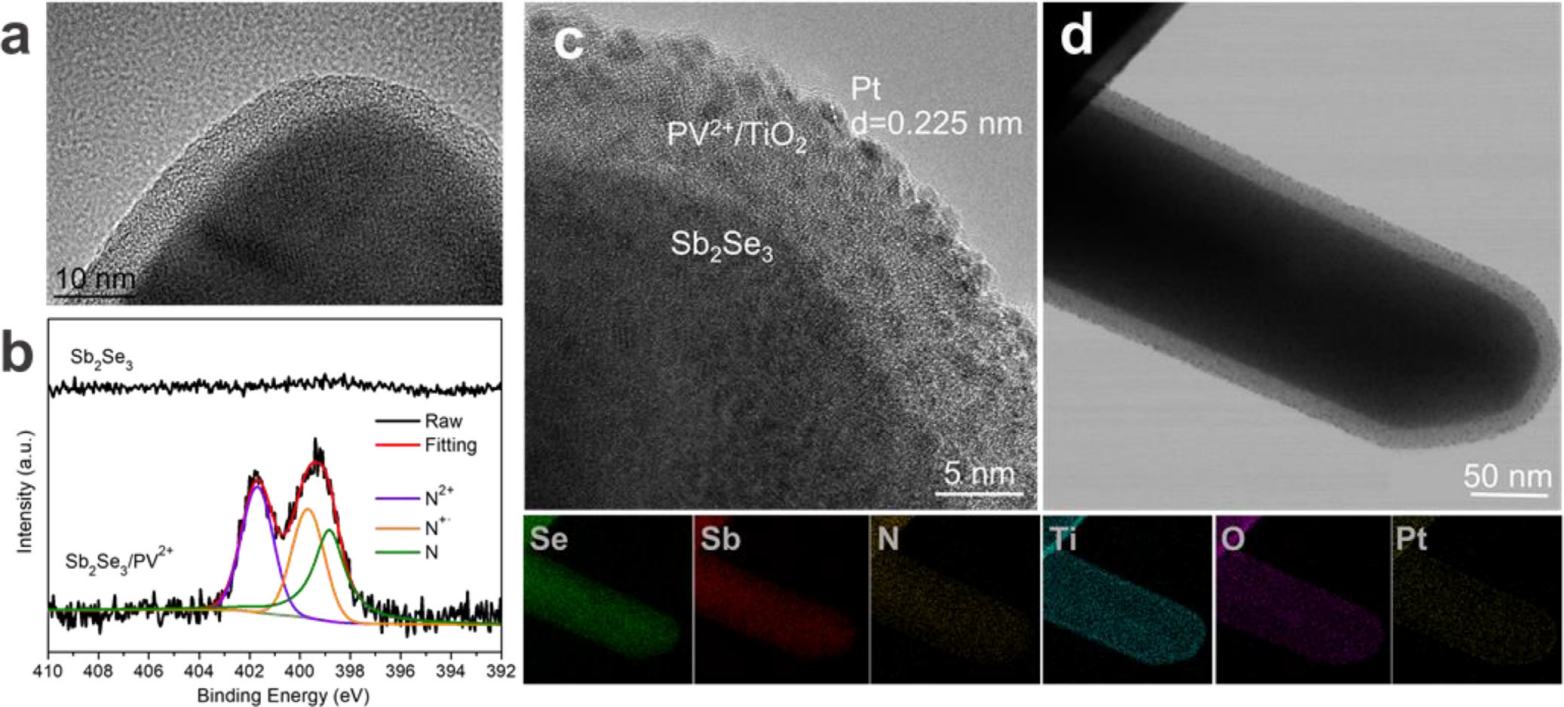
(a) HRTEM image of Sb_2_Se_3_/PV^2+^, (b) XPS of N 1S core level for Sb_2_Se_3_ and Sb_2_Se_3_/PV^2+^ thin films, (c) TEM image and corresponding EDS elemental mapping of Sb_2_Se_3_/PV^2+^/TiO_2_/Pt, elements detected: Se, Sb, N, Ti, O, and Pt; (d) HRTEM image of Sb_2_Se_3_/PV^2+^/TiO_2_/Pt.

A viologen derivative with allyl groups (1,1′-diallyl-[4,4-bipyridine]-1,1′-diium, the structure of which is shown in Scheme 1) was synthesized (see Support Information, Figs.S9 to S12, for details). The vinyl groups of V^2+^ can provide active sites for polymerization [32]. Thus, a photo-electropolymerization method was used to construct a viologen containing polymer (poly-1,1′-diallyl-[4,4-bipyridine]-1,1′-diium, herein PV^2+^) film on the surface of Sb_2_Se_3_ nanorods (termed Sb_2_Se_3_/PV^2+^). No obvious difference in the surface morphology between Sb_2_Se_3_/PV^2+^ and bare Sb_2_Se_3_ can be observed (Fig. S13). The HRTEM images of Sb_2_Se_3_/PV^2+^ (Fig. 1a) revealed a 5 nm thick amorphous layer on the surface of Sb_2_Se_3_ via photo-electropolymerization. As shown in Fig. S14, the TEM images and corresponding EDS analysis of Sb_2_Se_3_/PV^2+^ demonstrated that N and C were uniformly distributed on the surfaces of Sb_2_Se_3_ nanorods. In the XRD spectra, no new diffraction peaks were observed for Sb_2_Se_3_/PV^2+^ compared to the bare Sb_2_Se_3_, indicating that the PV^2+^ thin film was amorphous (Fig. S15). The presence of viologen on the surface of the Sb_2_Se_3_/PV^2+^ film was further confirmed by XPS. When PV^2+^ layer was introduced, N was clearly visible on the surface of Sb_2_Se_3_/PV^2+^ (Fig. S16), whereas no N was present on the bare Sb_2_Se_3_ surface. The high-resolution N 1s XPS spectrum of Sb_2_Se_3_/PV^2+^ could be fitted with three peaks (Fig. 1b). The binding energy of 401.7 eV was assigned to the electropositive pyridine nitrogen (-N^+^) of the viologen [33]. Meanwhile, binding energies of 399.7 and 398.8 eV were assigned to the radical cation nitrogen (-N^+•^) and neutral nitrogen (-N=) of viologen, respectively, which formed during XPS scanning [33]. The above characterization strongly suggests that an amorphous PV^2+^ layer was successfully introduced onto the surface of Sb_2_Se_3_ through the photo-electropolymerization.

A TiO_2_ layer was coated onto the surface of Sb_2_Se_3_ and Sb_2_Se_3_/PV^2+^ films by atomic layer deposition (ALD). Then, through a photo-assisted electrodeposition, Pt nanoparticles were deposited onto the corresponding electrodes as a hydrogen-generation catalyst, resulting in the fabrication of Sb_2_Se_3_/TiO_2_/Pt and Sb_2_Se_3_/PV^2+^/TiO_2_/Pt photocathodes. After TiO_2_ and Pt were deposited onto these photoelectrodes, the nanorod structures of Sb_2_Se_3_ and Sb_2_Se_3_/PV^2+^ were well maintained (Figs. S17-S19). Moreover, ultra-small nanoparticles could be observed on the surfaces of these nanorods. HRTEM images showed that Sb_2_Se_3_/PV^2+^/TiO_2_/Pt and Sb_2_Se_3_/TiO_2_/Pt both had a core-shell nanorod structure (Figs. 1c and S20a), with crystalline Sb_2_Se_3_ as the cores and the amorphous PV^2+^/TiO_2_ or TiO_2_ layer as the shell, and the ultra-small nanoparticles on the surfaces of these amorphous layers; the d-spacing of the adjacent fringe was 0.225 nm (assigned to the (111) crystalline plane of Pt lattice) [34]. TEM images and corresponding EDS analysis (Figs. 1d and S20b) clearly revealed that Se and Sb were distributed in the cores of nanorods; Ti, O and Pt were mainly distributed in the shell layer; and N (from PV^2+^) was present between the Sb_2_Se_3_ and TiO_2_ layers, confirming the successful construction of the Sb_2_Se_3_/PV^2+^/TiO_2_/Pt layer-by-layer core-shell structure.

**Fig. 2:**
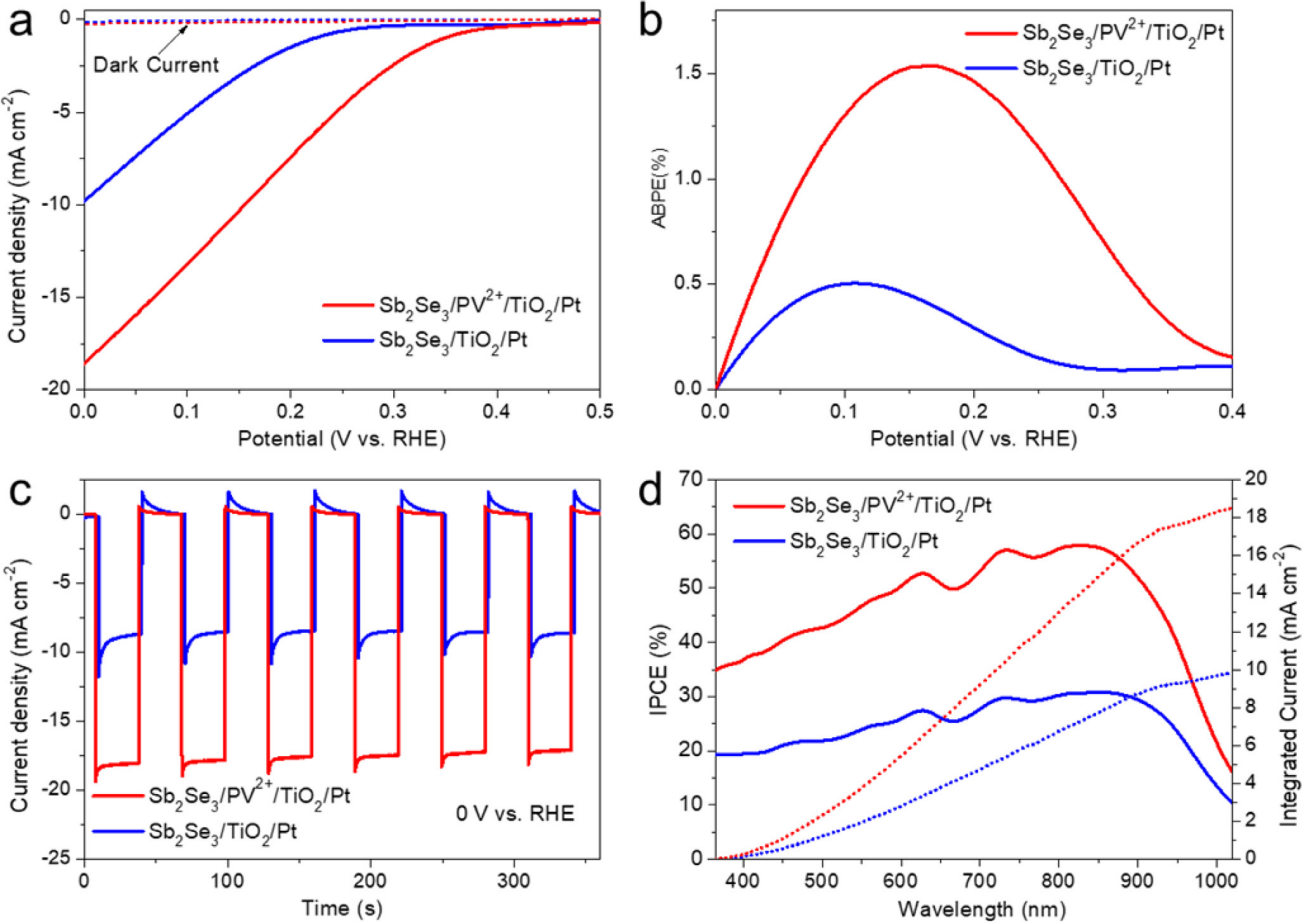
For Sb_2_Se_3_/TiO_2_/Pt and Sb_2_Se_3_/PV^2+^/TiO_2_/Pt photocathodes, (a) linear sweep voltammetry (LSV) curves under illumination (100 mW cm^−2^, AM 1.5G) in a 0.1 M Ar-purged H_2_SO_4_ electrolyte at a scan rate of 10 mV s^−1^; (b) applied bias photon-to-current efficiencies (ABPEs) calculated by LSV curves; (c) current-time (*I–t)* curves at 0 V vs. RHE under chopped illumination; and (d) incident photon-to-current efficiencies (IPCEs) measured at 0 V vs. RHE.

The photoelectrochemical performances of the Sb_2_Se_3_/TiO_2_/Pt and Sb_2_Se_3_/PV^2+^/TiO_2_/Pt photocathodes were recorded by using LSV curves. Sb_2_Se_3_/TiO_2_/Pt photocathode displayed a photocurrent density of −9.8 mA cm^−2^ at 0 V vs. RHE, with an onset potential of approximately 0.25 V vs. RHE (Fig. 2a). When the viologen ETM-containing polymer was inserted between Sb_2_Se_3_ and TiO_2_, a higher photocurrent density of −18.6 mA cm^−2^ (0 V vs. RHE) was achieved; this represents a remarkable performance enhancement (1.9 times). Meanwhile, the onset potential of the Sb_2_Se_3_/PV^2+^/TiO_2_/Pt photocathode positively shifted to 0.41 V vs. RHE compared to that of Sb_2_Se_3_/TiO_2_/Pt. A maximum ABPE (corresponding to half-cell solar-to-hydrogen conversion efficiency, HC-STH) of 1.54% at 0.17 V vs. RHE was achieved for Sb_2_Se_3_/PV^2+^/TiO_2_/Pt (Fig. 2b) according to the LSV data (Fig. 2a), which is approximately three times higher than that of Sb_2_Se_3_/TiO_2_/Pt photocathode without the PV^2+^ layer (0.51% at 0.10 V vs. RHE).

*I-t* curves of Sb_2_Se_3_/TiO_2_/Pt and Sb_2_Se_3_/PV^2+^/TiO_2_/Pt were measured under chopped illumination at a constant applied potential of 0 V vs. RHE (Fig. 2c). Photogenerated electron-hole pairs separated rapidly in the space charge region when the photocathodes were illuminated. Then the electrons moved to the photocathode surface, and charged the space capacitance, resulting in an ‘instantaneous’ photocurrent. The subsequent photocurrent decay is attributed to the recombination of the build-up of photogenerated electrons with the holes at or near the surface. Therefore, transient spikes can be observed, which could reveal the recombination process of photogenerated carriers. Obviously, Sb_2_Se_3_/PV^2+^/TiO_2_/Pt exhibited smaller transient spikes than Sb_2_Se_3_/TiO_2_/Pt, suggesting that the surface charge transfer of Sb_2_Se_3_/PV^2+^/TiO_2_/Pt was more effective by the presence of the PV^2+^ layer [35]. Meanwhile, photocurrent densities (0 V vs. RHE) of −9.3 and −18.4 mA cm^−2^ were obtained for Sb_2_Se_3_/TiO_2_/Pt and Sb_2_Se_3_/PV^2+^/TiO_2_/Pt, respectively, consistent with the LSV data shown in Fig. 2a. In addition, LSV curves of Sb_2_Se_3_/TiO_2_/Pt and Sb_2_Se_3_/PV^2+^/TiO_2_/Pt photocathodes under chopped light are shown in Fig. S21, the ‘transient current spikes’ were stronger over the entire applied bias for Sb_2_Se_3_/TiO_2_/Pt than that for Sb_2_Se_3_/PV^2+^/TiO_2_/Pt, especially near onset potential, which also suggested the PV^2+^ layer can effectively suppress recombination in bulk. Under a constant applied bias of 0.17 V vs. RHE, Sb_2_Se_3_/PV^2+^/TiO_2_/Pt retained ≈70% of its initial photocurrent (−10 mA cm^−2^) after 2 hours of continuous irradiation (Fig. S22), indicating good long-term stability. During the stability test, the amount of photoelectrochemically generated H_2_ was measured, showing a high Faradaic efficiency of ∼90% (Fig. S23).

IPCE of Sb_2_Se_3_/TiO_2_/Pt and Sb_2_Se_3_/PV^2+^/TiO_2_/Pt photocathodes were measured at a constant applied potential (0 V vs. RHE) under monochromatic light irradiation (Fig. 2d). Both Sb_2_Se_3_-based photocathodes could respond to the spectral region up to 1000 nm, in correspondence with the ultraviolet-visible (UV-vis) absorbance spectra (Fig. S24). In addition, the IPCEs of Sb_2_Se_3_/PV^2+^/TiO_2_/Pt were consistently higher than those of Sb_2_Se_3_/TiO_2_/Pt over the entire spectral region. A maximum IPCE of 57.9% was obtained for Sb_2_Se_3_/PV^2+^/TiO_2_/Pt at 825 nm, whereas for Sb_2_Se_3_/TiO_2_/Pt, the maximum IPCE was only 30.7%. Moreover, by integrating the IPCE data over the AM 1.5G solar spectrum (ASTM G173-03), integrated photocurrent densities of −9.8 and −18.5 mA•cm^−2^ (at 0 V vs. RHE) were acquired for the Sb_2_Se_3_/TiO_2_/Pt and Sb_2_Se_3_/PV^2+^/TiO_2_/Pt photocathodes, respectively, which are in line with LSV data (Fig. 1a). Integral saturation photocurrents of −9.7 and −18.5 mA•cm^−2^ at 0 V vs. RHE were obtained using the light spectrum of the solar simulator (Fig. S25), which is consistent with the integrated photocurrent densities over AM 1.5G solar spectrum.

Complex physical and electrochemical processes are known to be involved in PEC water splitting systems. Thus, an in-depth mechanistic understanding would be indispensable for the rational design and development of highly efficient PEC cells [36]. A typical photocathode for PEC H_2_ production involves three main steps: generating charge carriers by exciting the semiconductor, migrating these charge carriers from the bulk to the surface of photocathode, and generating H_2_ on the surface catalytic sites. As Sb_2_Se_3_/PV^2+^/TiO_2_/Pt and Sb_2_Se_3_/TiO_2_/Pt both share the same light-harvesting material (Sb_2_Se_3_) and catalyst (Pt), the significant difference in their PEC performances can be attributed to the efficient interfacial charge separation and transfer promoted by the PV^2+^ ETM layer.

**Fig. 3:**
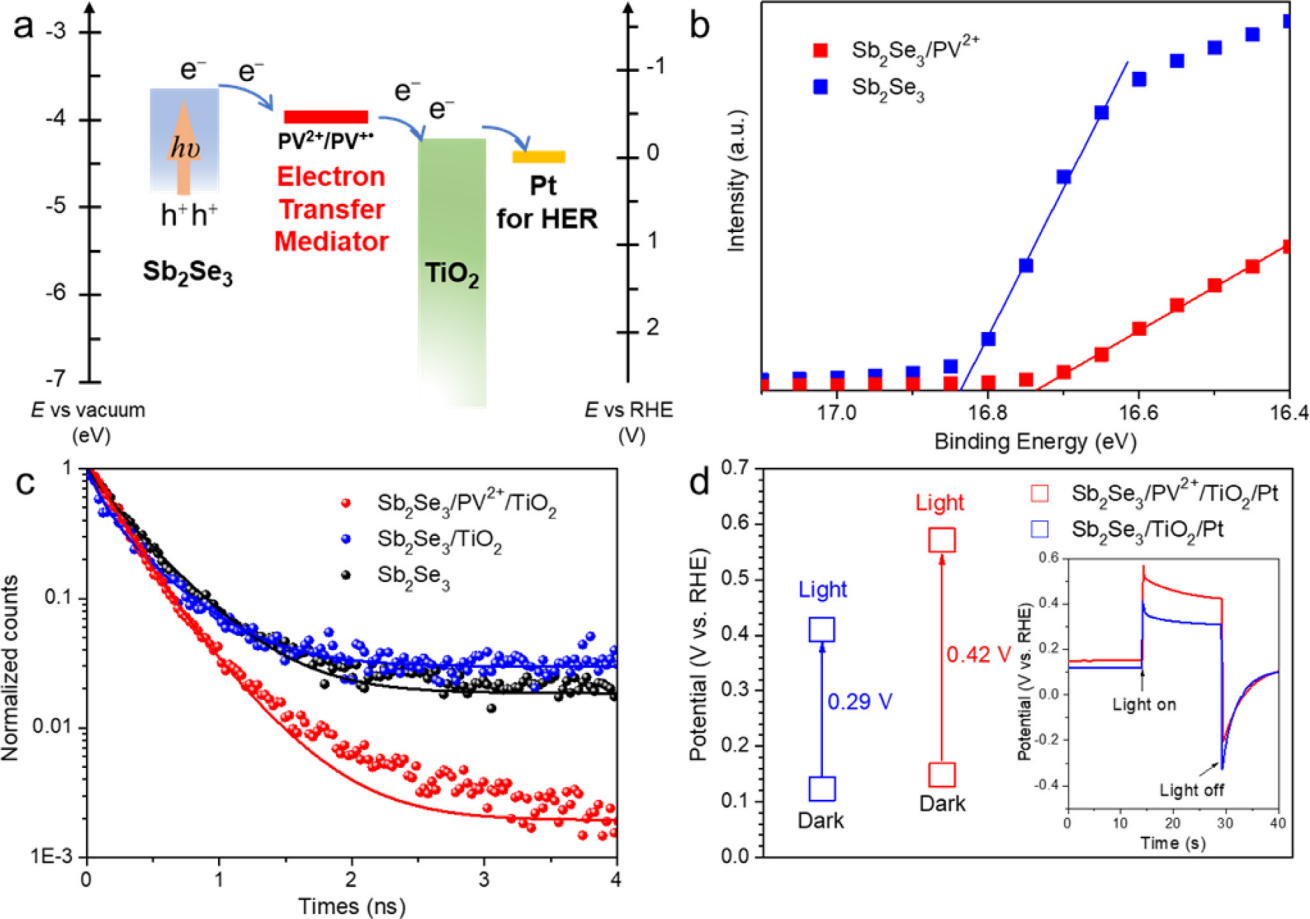
(a) The electron energy profile of the *E*_CB_ for Sb_2_Se_3_ and TiO_2_; the redox potential of PV^2+^ and overpotential of Pt for HER. (b) Ultraviolet photoemission spectroscopy (UPS) electron cutoff regions for Sb_2_Se_3_ and Sb_2_Se_3_/PV^2+^ films, solid lines represent linear regression to spectral cutoff feature. (c) Time-resolved photoluminescence (TR-PL) emission decay spectra of Sb_2_Se_3_, Sb_2_Se_3_/TiO_2_, Sb_2_Se_3_/PV^2+^/TiO_2_ films. (d) Open-circuit potential (OCP) measurements under both dark and light conditions for Sb_2_Se_3_/TiO_2_/Pt and Sb_2_Se_3_/PV^2+^/TiO_2_/Pt.

To confirm this inference, the energy-band alignments of Sb_2_Se_3_, PV^2+^, and TiO_2_ were estimated. The band position of Sb_2_Se_3_ was measured according to UV-vis absorbance spectra and UV photoemission spectroscopy (UPS) (Fig. S26). And the band positions of TiO_2_ were estimated according to the flat-band potential (*E*_fb_, Mott−Shockley plots) and UV-vis absorbance spectra of TiO_2_ (Fig. S27 and S28). The results revealed that the as-prepared Sb_2_Se_3_ had a conduction band energy (*E*_CB_) of -3.73 eV (−0.71 V vs. RHE) and a valence band energy (*E*_VB_) of −4.86 eV (0.42 V vs. RHE) with a bandgap of 1.13 eV, and TiO_2_ has an *E*_CB_ of −4.22 eV (−0.22 V vs. RHE), and an *E*_VB_ of −7.72 eV (3.28 V vs. RHE). The redox potential of viologen in the PV^2+^ layer was estimated using a cyclic voltammogram (CV) and the corresponding differential pulse voltammogram (DPV) of the monomer of methyl viologen (MV^2+^) in 0.1 M H_2_SO_4_ (Fig. S29), showing the reversible redox of V^2+^/V^+•^ at −0.38 V vs. RHE. The onset potential of Pt for hydrogen evolution is near 0 V vs. RHE (Fig. S30). According to the above data, the band alignment diagrams of Sb_2_Se_3_, PV^2+^, TiO_2_ and Pt are shown in Fig. 3a. A conduction band (CB) offset of ∼0.2 eV is desirable for high-quality heterojunctions [37], [38]. Obviously, a large conduction band offset (∼0.48 eV) was predicted for the Sb_2_Se_3_/TiO_2_ junction; such a high energy band alignment would form a large cliff-like barrier for electron transport [[20], [21], 39]. In other words, photogenerated electrons would have to overcome a large energy barrier when migrating from Sb_2_Se_3_ to TiO_2_, which would provide these photoelectrons with more opportunities to recombine with holes, while reducing their chances of participating in the desired chemical reaction at the electrode's surface [19]. When the viologen-containing PV^2+^ layer was introduced into the Sb_2_Se_3_/TiO_2_ heterojunction interface, the electrons photogenerated at Sb_2_Se_3_ could smoothly transfer to the viologens (V^2+^) and react with the corresponding cationic radicals (V^+•^). Meanwhile, the viologen radicals (V^+•^) could also readily inject electrons into the CB of TiO_2_ and regenerate the viologens (V^2+^). Thus, the redox potential of V^2+^/V^+•^ monomer within the viologen-containing polymer was suitable for placement between the conduction band of Sb_2_Se_3_ and TiO_2_, which could adjust the band alignment, thereby promoting the interfacial carrier transport by serving as an ETM.

UPS measurements were performed to determine the band alignment change experienced when PV^2+^ was introduced. As shown in Fig. 3b, the cutoff of the secondary electron of Sb_2_Se_3_/PV^2+^ (16.72 eV) shifted to the lower binding energy of 0.11 eV, compared to that of Sb_2_Se_3_ (16.83 eV), suggesting that the Fermi level of Sb_2_Se_3_/PV^2+^ was more anodic than that of Sb_2_Se_3_ [40]. As shown in Fig. S31a, this Fermi level shift would cause the energy level of the conduction band (*E*_cb_) of a Sb_2_Se_3_-based semiconductor to be closer to the CB of TiO_2_ under the flat-band conditions (or natural alignments, in reference to the vacuum level). This would result in a larger internal potential between the interface of Sb_2_Se_3_ and TiO_2_, compared to that of Sb_2_Se_3_/TiO_2_. Correspondingly, under equilibrium conditions, a larger band bending energy (*E*_b_) would be generated between the interface of Sb_2_Se_3_ and TiO_2_ after the insertion of the PV^2+^ ETM layer (Fig. S31b). A large *E*_b_ can strongly improve the interfacial charge separation, thereby effectively reducing the charge recombination. This promotes charge transfer, thus improving the PEC performance of the resulting devices [41].

To further elucidate the function of the PV^2+^ ETM layer, and the charge recombination properties between Sb_2_Se_3_ and TiO_2_, with/without the assistance of the PV^2+^ ETM layer, time-resolved photoluminescence (TR-PL) analysis was conducted without a catalyst (Fig. 3c). A pump wavelength of 470 nm was selected, whose energy is above the bandgap of Sb_2_Se_3_, so that photogenerated carriers could be produced directly. The lifetimes of recombination for the photoexcited holes and electrons could be estimated by measuring the signal of luminescence decay caused by recombination [42]. The TR-PL decay kinetics of Sb_2_Se_3_, Sb_2_Se_3_/TiO_2_ and Sb_2_Se_3_/PV^2+^/TiO_2_ were fitted with the double exponential function, where the τ_1_ and τ_2_ correspond to the fast and slow lifetime components, respectively (Table S1) [42]. Similar values for τ_1_ (0.39, 0.41 and 0.39 ns) were obtained for Sb_2_Se_3_, Sb_2_Se_3_/TiO_2_ and Sb_2_Se_3_/PV^2+^/TiO_2_, respectively, which could be considered as the non-radiative decay caused by the trap states of Sb_2_Se_3_, and independent to the presence of the carriers acceptor layers [43]. However, τ_2_ is related to radiative interband recombination dynamics, which decrease if a contact layer can extract photogenerated carriers [42]. The similar τ_2_ values (0.22 and 0.21 ns) were obtained for Sb_2_Se_3_ and Sb_2_Se_3_/TiO_2_, suggesting that TiO_2_ could not effectively suppress the charge recombination. Meanwhile, τ_2_ decreased from 0.21 to 0.12 ns after the PV^2+^ layer was introduced to the Sb_2_Se_3_/TiO_2_ interface, suggesting that the photoelectrons generated by Sb_2_Se_3_ could be more efficiently extracted with the PV^2+^ layer. These findings are consistent with the above conclusion that introducing the PV^2+^ ETM layer would result in a larger *E*_b_, thereby effectively reducing the charge recombination.

The PL lifetime directly correlates with the open circuit potential (OCP), which is an important indicator for thin-film devices [44]. The photovoltage at the photocathode/electrolyte interface was determined using OCP measurements, which were implemented under both dark (*V*_dark_) and light (*V*_light_) conditions with a zero net exchange current (Fig. 3d). In this way, thermodynamic information was collected while minimizing the influences of kinetic factors [45]. Ideally, the *V*_dark_ of a photoelectrode in contact with an aqueous electrolyte should equal the thermodynamics potential of the corresponding redox reaction, with a potential drop (*η*) determined by a given catalyst [45]. With Pt nanoparticles as the catalyst, the *V*_dark_ of Sb_2_Se_3_/PV^2+^/TiO_2_/Pt (0.15 V) was similar to that of Sb_2_Se_3_/TiO_2_/Pt (0.12 V). This similarity arose from the fact that the same catalyst was employed for both photocathodes. The Sb_2_Se_3_/PV^2+^/TiO_2_/Pt exhibited a much higher *V*_light_ (0.57 V vs. RHE) than Sb_2_Se_3_/TiO_2_/Pt (0.41 V vs. RHE). The corresponding photovoltage (*V*_ph_) values were calculated as 0.29 and 0.42 V for Sb_2_Se_3_/TiO_2_/Pt and Sb_2_Se_3_/PV^2+^/TiO_2_/Pt, respectively. Thus, introducing the PV^2+^ ETM layer produced a higher *V*_ph_ at the photocathode-electrolyte interface, which is advantageous for the suppression of the charge recombination process and the acceleration of surface charge transfer process, resulting in a more positive shift in the onset potential and a higher photocurrent density [41].

The transient photocurrent response has also been obtained using chronoamperometry to further investigate the charge recombination behavior of Sb_2_Se_3_/PV^2+^/TiO_2_/Pt and Sb_2_Se_3_ /TiO_2_/Pt, at a bias of 0 V vs. RHE (Fig. S32 inset). The photo-response with respect to the ON/OFF cycle was faster for the Sb_2_Se_3_/PV^2+^/TiO_2_/Pt photocathode than for Sb_2_Se_3_ /TiO_2_/Pt. Correspondingly, normalized plots of the natural logarithm for a normalized parameter (*D*) extracted from transient photocurrent curves as a function of time (*t*) were plotted (Fig. S32), where the time at ln *D* = −1 was defined as a transient time constant (*T*) [46]. *T* was calculated to be 1.89 and 3.91 s for Sb_2_Se_3_/TiO_2_/Pt and Sb_2_Se_3_/PV^2+^/TiO_2_/Pt, respectively. To some extent, the larger *T* of the latter confirmed that charge recombination was suppressed following the introduction of the PV^2+^ layer at the Sb_2_Se_3_/TiO_2_ interface.

**Fig. 4:**
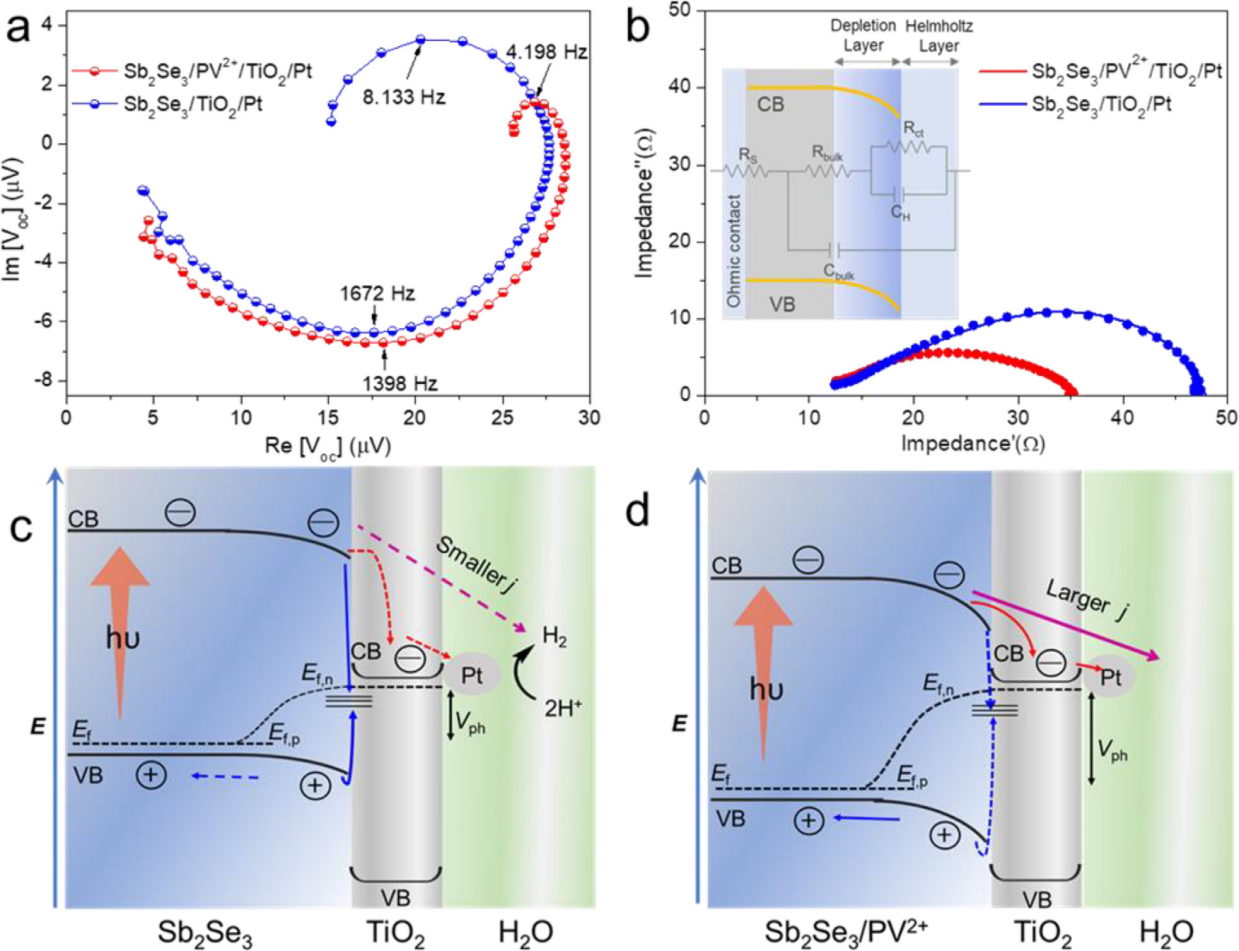
(a) IMVS spectra of Sb_2_Se_3_/TiO_2_/Pt and Sb_2_Se_3_/PV^2+^/TiO_2_/Pt photocathodes. (b) Nyquist plots from PEIS experiments, fitting curves are shown by solid lines, and the inset is the equivalent circuit. Simplified schemes of elementary processes in (c) Sb_2_Se_3_/TiO_2_/Pt and (d) Sb_2_Se_3_/PV^2+^/TiO_2_/Pt photocathodes. Movements of electrons generated from photo-induced charge separation show main pathways (solid lines: majority events; dotted lines: minority events).

As a powerful technique for analyzing the photovoltage of a photoelectrode, intensity-modulated photovoltage spectroscopy (IMVS) offers insights into the charge recombination kinetics under open-circuit conditions [47], [48]. The measured IMVS response of the Sb_2_Se_3_/TiO_2_/Pt and Sb_2_Se_3_/PV^2+^/TiO_2_/Pt photocathodes are depicted in Fig. 4a, where two semicircles could be observed in the low-frequency (quadrant I) and high-frequency (quadrant IV) region, respectively. This high-frequency semicircle has often been observed in prior studies; it is linked to the recombination processes in the bulk of photocathode [47]. The frequency at the minimum point of the Nyquist plot (*f*_min_) relates to the lifetimes of the photogenerated charge in the space charge region (*τ_n_*), which can be calculated by *τ*_n_ = 1/2π*f*_min_, which reflects recombination occurring in the bulk electrode [49]. As shown in Table S2, a *f*_min_ of 1672 Hz with a *τ*_n_ of 95.2 μs was obtained for Sb_2_Se_3_/Pt. In contrast, a superior *τ*_n_ value of 113.9 μs was estimated from the *f*_min_ of 1398 Hz after the introduction of the PV^2+^ ETM layer. The larger *τ*_n_ of the latter indicates that adding PV^2+^ ETM layer may have suppressed the charge recombination at the Sb_2_Se_3_/TiO_2_ interface, as longer lifetimes of photogenerated carriers correspond to the greater time required for their separation and transportation. This longer time period is favorable for the collection of photogenerated carriers. Similarly, the time constant associated with surface recombination (*τ*_int_; *τ*_int_=1/2π*f*_max_) can be estimated using the frequency at the maximum point (*f*_max_) [47]. The *τ*_n_ value obtained for Sb_2_Se_3_/PV^2+^/TiO_2_/Pt (37.9 ms) was almost twice that obtained for Sb_2_Se_3_/TiO_2_/Pt (19.5 ms). This increase in the lifetimes of the photogenerated carriers indicates that the PV^2+^ ETM layer could suppress bulk and surface charge recombination.

Photoelectrochemical impedance spectroscopy (PEIS) was carried out to investigate the charge transfer phenomena, both in bulk and at the photocathode/electrolyte interface. The PEIS spectra of the Sb_2_Se_3_/TiO_2_/Pt and Sb_2_Se_3_/PV^2+^/TiO_2_/Pt photocathodes were fitted by the Randles equivalent circuit, as shown in Fig. 4b, which included bulk resistance (R_bulk_), charge-transfer resistance (R_ct_) at the photocathode/electrolyte interface, series resistance (R_s_), space charge region capacitance (C_bulk_), and surface-state capacitance (C_H_) in Helmholtz layer [50]. The corresponding calculated values of these parameters are listed in Table S3. In the Nyquist plots, the charge transport behavior is roughly reflected by the radius of each arc. For Sb_2_Se_3_/PV^2+^/TiO_2_/Pt, there was a smaller-radius arc, with a lower R_ct_ and a higher C_H_ (5.88 Ω and 11.1 μF, respectively), indicating that the charge transfer process was faster at the photocathode/electrolyte interface than that for Sb_2_Se_3_/TiO_2_/Pt (13.7 Ω and 7.89 μF, respectively). In addition, the values of R_bulk_ decreased significantly (from 23.9 to 18.9 Ω when PV^2+^ was added to the system, while C_bulk_ increased from 163 to 216 μF. The above results demonstrate that the charge transport process in bulk was faster compared to Sb_2_Se_3_/TiO_2_/Pt, especially from Sb_2_Se_3_ layer to TiO_2_ layer. Therefore, the introduction of the PV^2+^ EMT layer into the Sb_2_Se_3_/TiO_2_ interface could accelerate the charge separation/transfer process, thus enhancing the PEC water-splitting performance.

The IMVS and PEIS data agreed well with the TR-PL, OCP and photocurrent density–time dependence results. Conceptually, *V*_ph_ is generated by the Fermi level splitting under illumination, which is the difference between the quasi-Fermi level of holes (*E*_F,p_) and the quasi-Fermi level of electrons (*E*_F,n_). Accordingly, the critical role of the PV^2+^ EMT layer in the photocathode is summarized Fig. 4c,d. Introducing the PV^2+^ EMT layer produces a higher *V*_ph_ at the photocathode–electrolyte interface and larger band bending. This could suppress the charge recombination and accelerate the surface charge transfer process, resulting in a more positive shift in the onset potential and a higher photocurrent density [41]. This is caused by the PV^2+^ EMT layer regulating the energy band alignment of the Sb_2_Se_3_/TiO_2_ interface by altering the Fermi level shift, thus bringing the energy level of the CB of Sb_2_Se_3_/PV^2+^ closer to that of TiO_2_. This results in a relatively small cliff-like barrier for electron transport. As a large cliff-like barrier exists between Sb_2_Se_3_ and TiO_2_, electrons injection from the CB of Sb_2_Se_3_ to that of TiO_2_ has to overcome a large energy gap, leading to a slow charge transport kinetics and serious levels of charge recombination. However, the photogenerated electrons from Sb_2_Se_3_ can be effectively transferred to TiO_2_ and rapidly consumed for H_2_ generation with the assistance of the PV^2+^ EMT layer. Correspondingly, these photogenerated electrons have less possibility to recombine with photogenerated holes at each interface, a higher photocurrent of Sb_2_Se_3_/PV^2+^/TiO_2_/Pt than that of Sb_2_Se_3_/TiO_2_/Pt.

## 4 Conclusion

In summary, a rational strategy was developed by introducing a PV^2+^ film between the interface of Sb_2_Se_3_ and TiO_2_. This PV^2+^ EMT layer could tune the energy band alignment of the Sb_2_Se_3_/TiO_2_ interface by inducing a Fermi level shift and decreasing the cliff-like barrier of the Sb_2_Se_3_/TiO_2_ interfacial electron transport process. This allowed photogenerated electrons from Sb_2_Se_3_ to be more effectively transferred to TiO_2_ for H_2_ generation. Therefore, Sb_2_Se_3_/PV^2+^/TiO_2_/Pt exhibited much better PEC performance than Sb_2_Se_3_/TiO_2_/Pt, including a higher *V*_ph_ at the photocathode−electrolyte interface, a superior photocurrent density (−18.6 mA•cm^−2^ at 0 V vs. RHE), a more positive onset potential (0.41 V vs. RHE), and a higher HC-STH (up to 1.54%). This study provides an in-depth understanding of the functions of viologen-containing ETM layers. These findings can contribute to the future development and application of promising and versatile organic-based ETMs in PEC systems, thereby helping to achieve efficient PEC water splitting.

## Declaration of competing interest

The authors declare that thay have no conflicts of interest in this work.

## Appendix Supplementary materials

Image, application 1
