# Supplementary material for: Polymeric viologen-based electron transfer mediator for improving the photoelectrochemical water splitting on Sb2Se3 photocathode
Source: Fundam Res. 2022 Apr 1;4(2):291–9. doi: 10.1016/j.fmre.2022.03.013 (PMC11197680; doi:10.1016/j.fmre.2022.03.013)
Supplement: Supplementary file 1 [file mmc1.docx]

Polymeric Viologen-Based Electron Transfer Mediator for Improving the Photoelectrochemical Water Splitting on Sb_2_Se_3_ Photocathode

*Chang Liu^a,1^, Fusheng Li^*,a,1^, Linqin Wang^b^, Zeju Li^a^, Yilong Zhao^a^, Yingzheng Li^a^, Wenlong Li^a^, Ziqi Zhao^a^, Ke Fan^a^, Fei Li^a^, Licheng Sun^a,b,c^*

^a^ State Key Laboratory of Fine Chemicals, Institute of Artificial Photosynthesis, DUT-KTH Joint Education and Research Centre on Molecular Devices, Institute for Energy Science and Technology, Dalian University of Technology, 116024 Dalian, China.

^b^ Center of Artificial Photosynthesis for Solar Fuels, School of Science, Westlake University, 310024 Hangzhou, China.

^c^ Department of Chemistry, School of Engineering Sciences in Chemistry, Biotechnology and Health, KTH Royal Institute of Technology, Stockholm 10044, Sweden.

*** Corresponding author: [fusheng@dlut.edu.cn](mailto:fusheng@dlut.edu.cn) (F. Li)

^1^ *These authors contributed equally to this work*

**Experience section**

**Instruments**. The surface morphology of the fabricated films was characterized by field emission scan electron microscopy (FE-SEM, HITACHI SU8220, operate at 5 kV) and field emission transmission electron microscopy (JEM-F200). The crystal structures were characterized by X-ray diffraction (XRD) with Cu Kα (1.54056 Å) radiation (SmartLab 9KW), and the surface compositions and the band diagrams of the electrode films were investigated using X-ray photoelectron spectroscopy (XPS) and (ultraviolet photoelectron spectroscopy (UPS) on an ESCALAB Xi^+^ (Thermo Scientific™). The chemical structure of the films was studied using Raman spectroscopy (LabRAM HR800, 532 nm excitation wavelength; HORIBA JobinYvon) and Fourier transform infrared spectrum (Thermo Fisher™, Nicolet 6700 Flex). NMR spectra were recorded on a Bruker Advance 500 spectrometer. Mass spectrometry measurements were performed on a Bruker MALDI-TOF spectrometer.

**Materials.** Antimony selenide (Sb_2_Se_3_, 99.99%)，allyl bromide (C_3_H_5_Br, 98%)，and 4,4’-bipyridine (98%) were purchased from Innochem Reagent, Beijing, China. Selenourea (CH_4_N_2_Se, 98%) and Dihydrogen hexachloroplatinate(IV) hexahydrate (H_2_Cl_6_O_4_·6H_2_O, 99%) were purchased from Alfa Reagent, Shanghai, China. All the reagents were used as received without further treatment. All aqueous solutions were prepared with high-purity deionized water (Milli-Q, resistance 18 MΩ cm^−1^).

**Characterization**

**Incident photon-to-current conversion efficiency (IPCE)** measurements of the Sb_2_Se_3_-based photocathodes were directly measured by CIMPS-2 (Zahner). The wavelength range was chosen from 365 to 1020 nm at a constant applied potential of 0 V vs. RHE.

**Time-resolved photoluminescence (TR-PL)** measurements were carried out on WITec alpha300RAS confocal Raman microscopy (WITec GmbH). The samples were excited with a picosecond pulsed diode laser (Pico-quant LDH 405), with a ∼ 70 ps pulse width and 20 MHz repetition rate, focused on the sample with a 100 x objective (NA=0.90). The PL signal was acquired through the time-correlated single-photon counting (TCSPC) StrobeLock system. The total instrument response function (IRF) for the PL decay was less than 200 ps, and the temporal resolution was less than 30 ps. To obtain the exact PL lifetime, all PL data were fitted as single exponential decay via WITec Project Plus software.

**Photoelectrochemical impedance spectroscopy (PEIS)** measurements were conducted by applying an AC voltage amplitude of 10 mV at 0 V vs. RHE under simulated AM 1.5G illumination (100 mW cm^-2^) within the frequency range from 50k to 0.1 Hz in 0.1 M H_2_SO_4_.

**Intensity-modulated photovoltage spectroscopy (IMVS)** was conducted by CIMPS-2 (Zahner) under the 100 mW/cm^2^ illumination with 10% superimposition of sinusoidal modulation. The frequency of modulation was swept from 100 kHz to 0.1 Hz.

**Fig. S1.** **The thickness test curve of Sb_2_Se_3_ thin film by a stylus profiler.**

**Fig. S2.**  **X-ray diffraction (****XRD) pattern of Sb_2_Se_3_ thin film.**

**Fig. S3.** **Raman scatter spectrum of Sb_2_Se_3_ thin film.**

**Fig. S4.** **X-ray photoelectron spectroscopy (XPS) survey spectrum of Sb_2_Se_3_ film.**


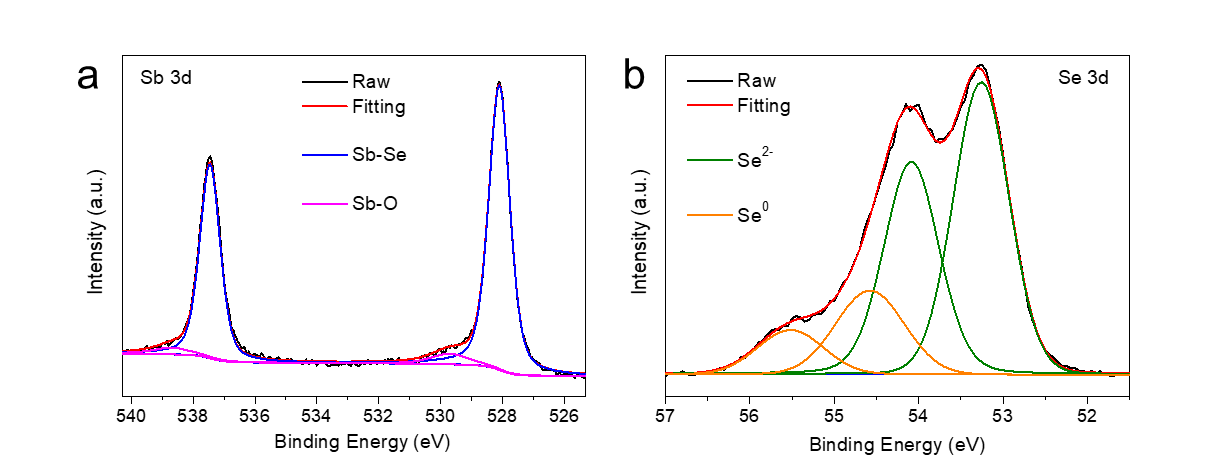


**Fig. S5.** **XPS of (a) Sb 3d core level and (b) Se 4d core level of Sb_2_Se_3_ thin film.**


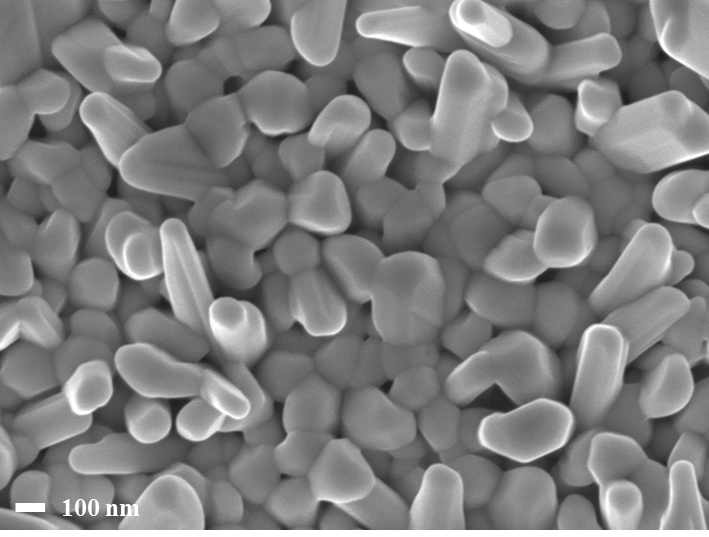


**Fig. S6. The surface image of scanning electron microscopy (SEM) for Sb_2_Se_3_.**


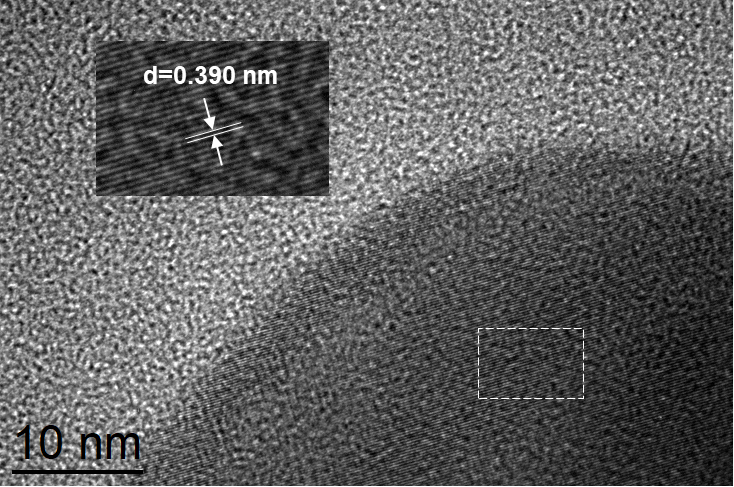


**Fig. S7.**  **High-resolution transmission electron microscopy (HRTEM) image for Sb_2_Se_3_, the inset showed the enlarged region.**

**
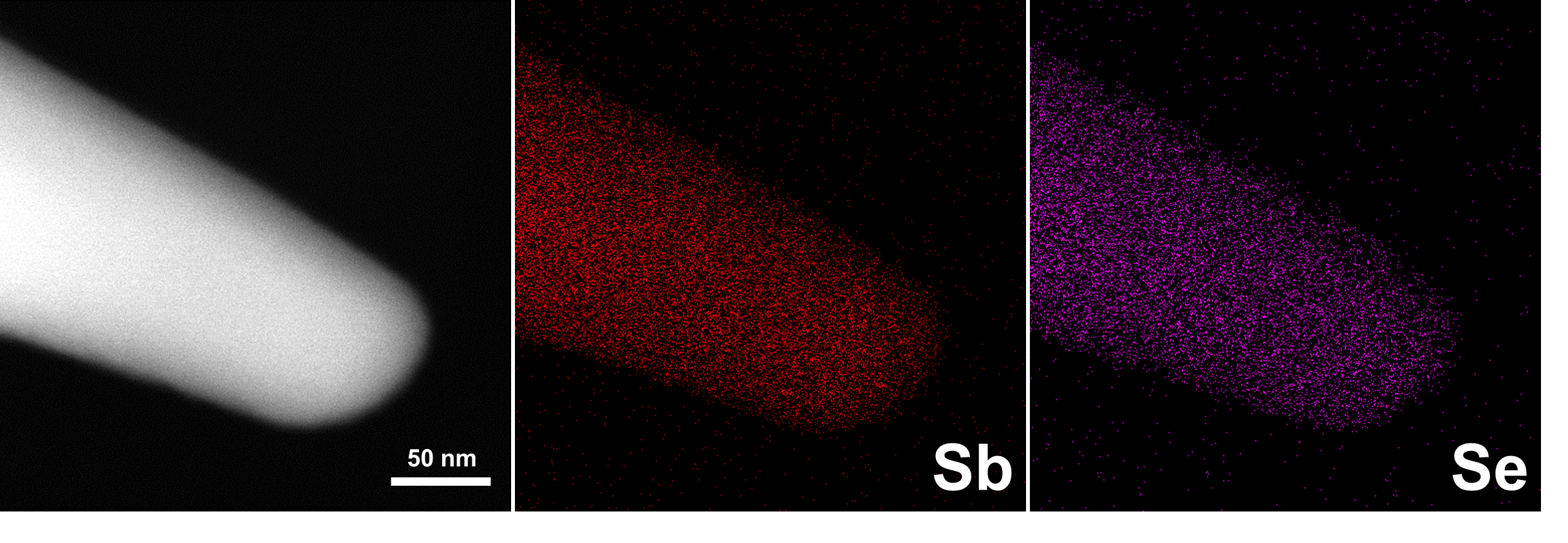
**

**Fig. S8.** **Transmission electron microscopy (TEM) image and EDS elemental mapping of the Sb_2_Se_3_ film. Elements detected: Se, Sb.**

**
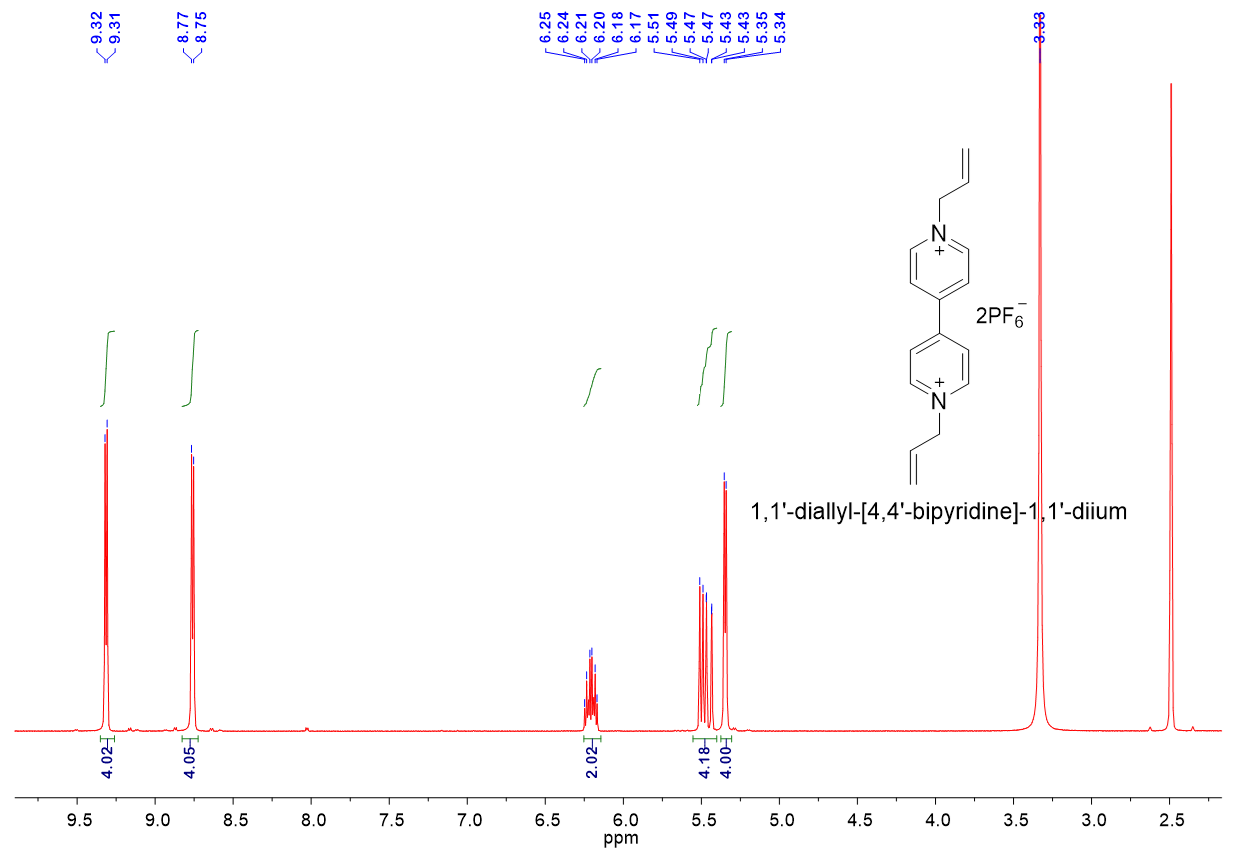
**

**Fig. S9.** **^1^H NMR spectrum (500 MHz, CD_3_CN) of PV^2+^.**


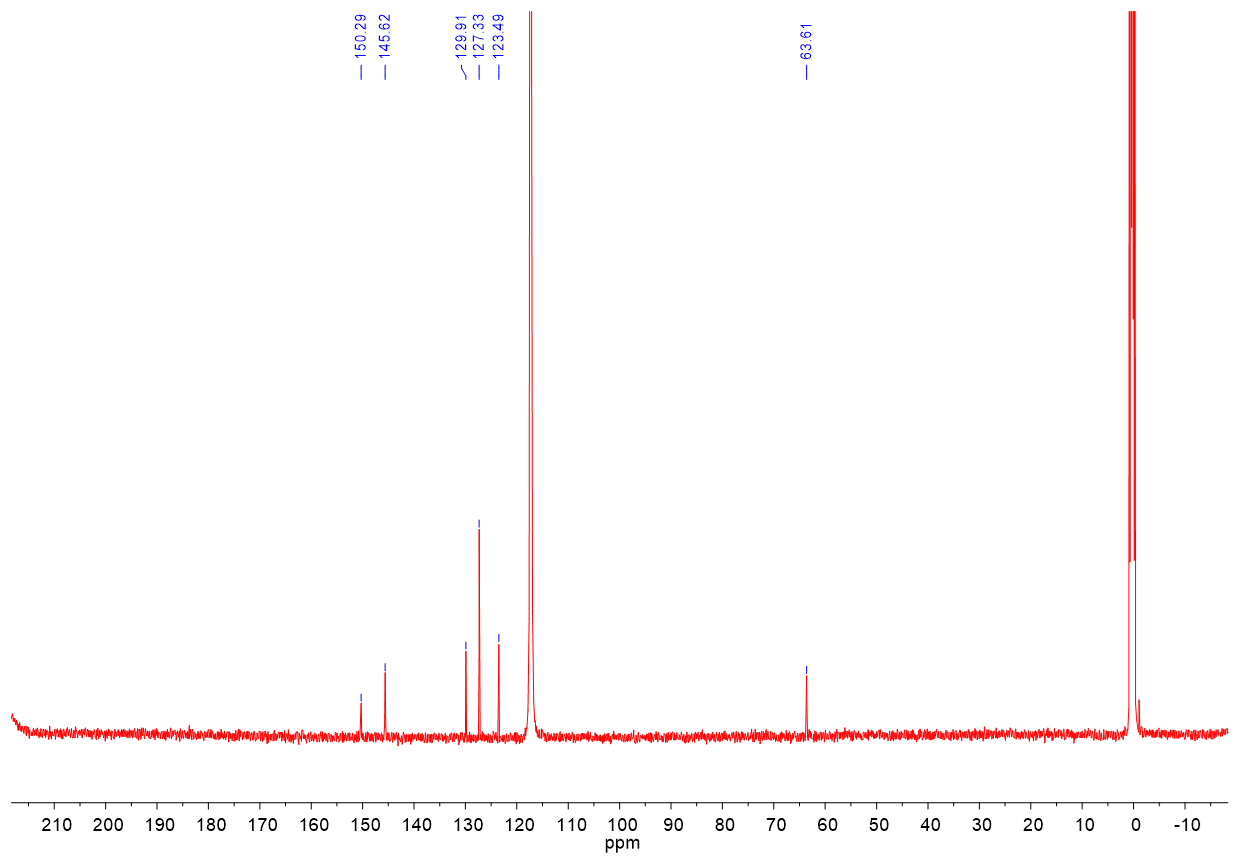


**Fig. S10.**  **^13^C NMR** **spectrum (500 MHz, CD_3_CN) of PV^2+^.**

^^

**Fig. S11.**  **Electrospray mass spectrum of PV^2+^.**

**Fig. S12.**  **Fourier transform infrared spectroscopy (FTIR) of PV^2+^.**


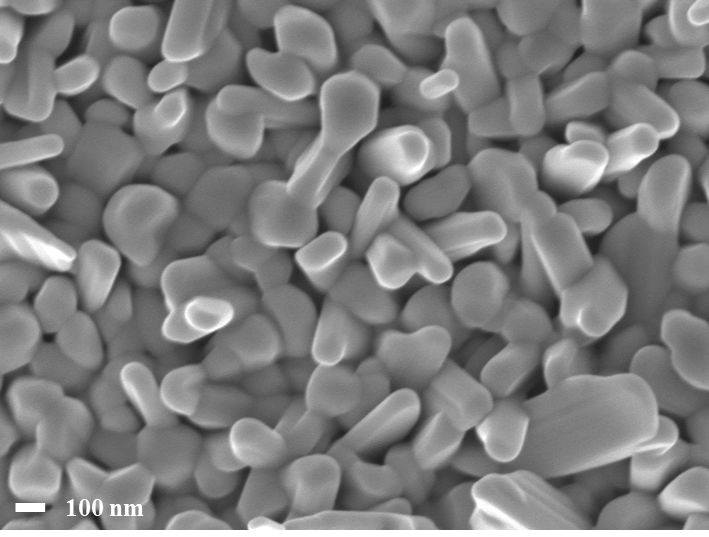


**Fig. S13.**  **SEM surface image for Sb_2_Se_3_/PV^2+^.**


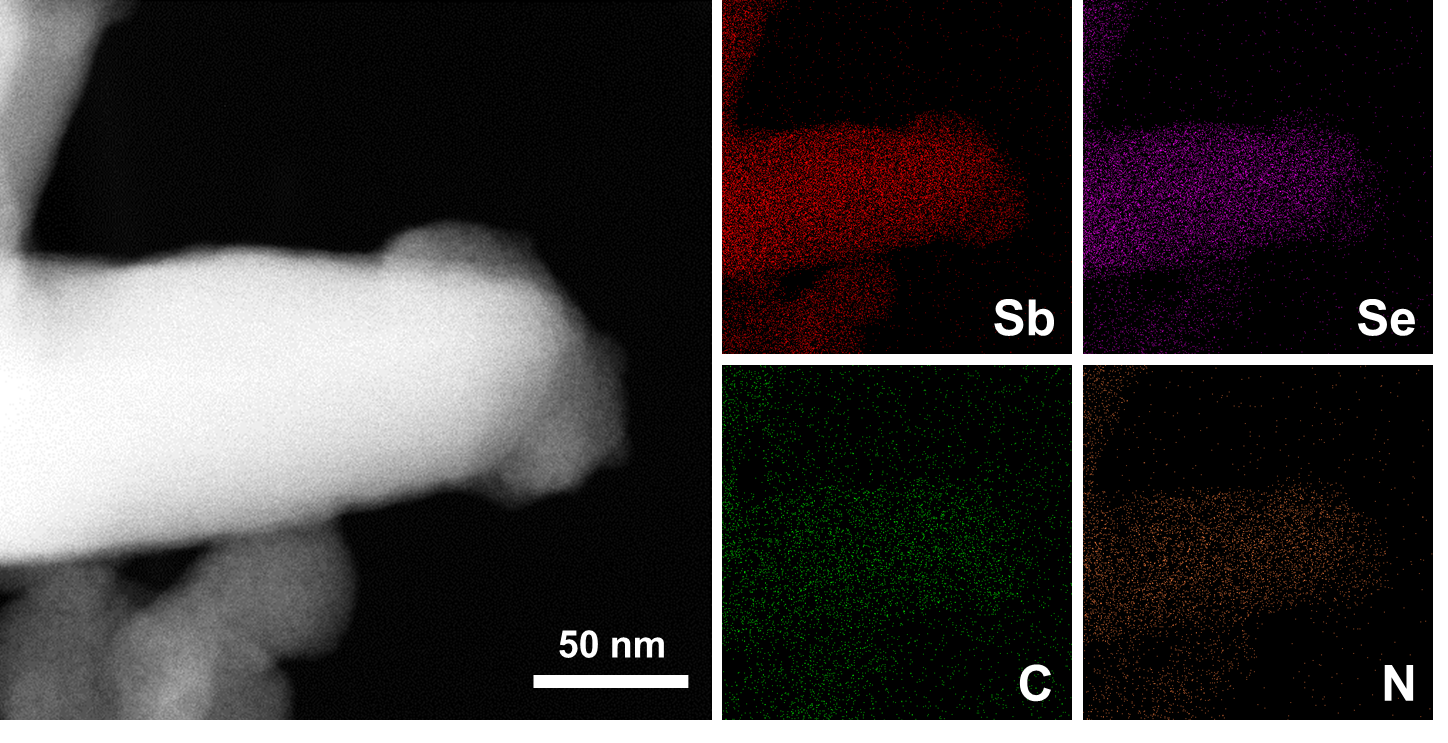


**Fig. S14.** **TEM image and EDS elemental mapping of the Sb_2_Se_3_/PV^2+^ film. Elements detected: Se, Sb, C, N**

**Fig. S15.** **XRD patterns of Sb_2_Se_3_ and Sb_2_Se_3_/PV^2+^ thin films.**

**Fig. S16.** **XPS survey spectrum of the Sb_2_Se_3_/PV^2+^ film.**


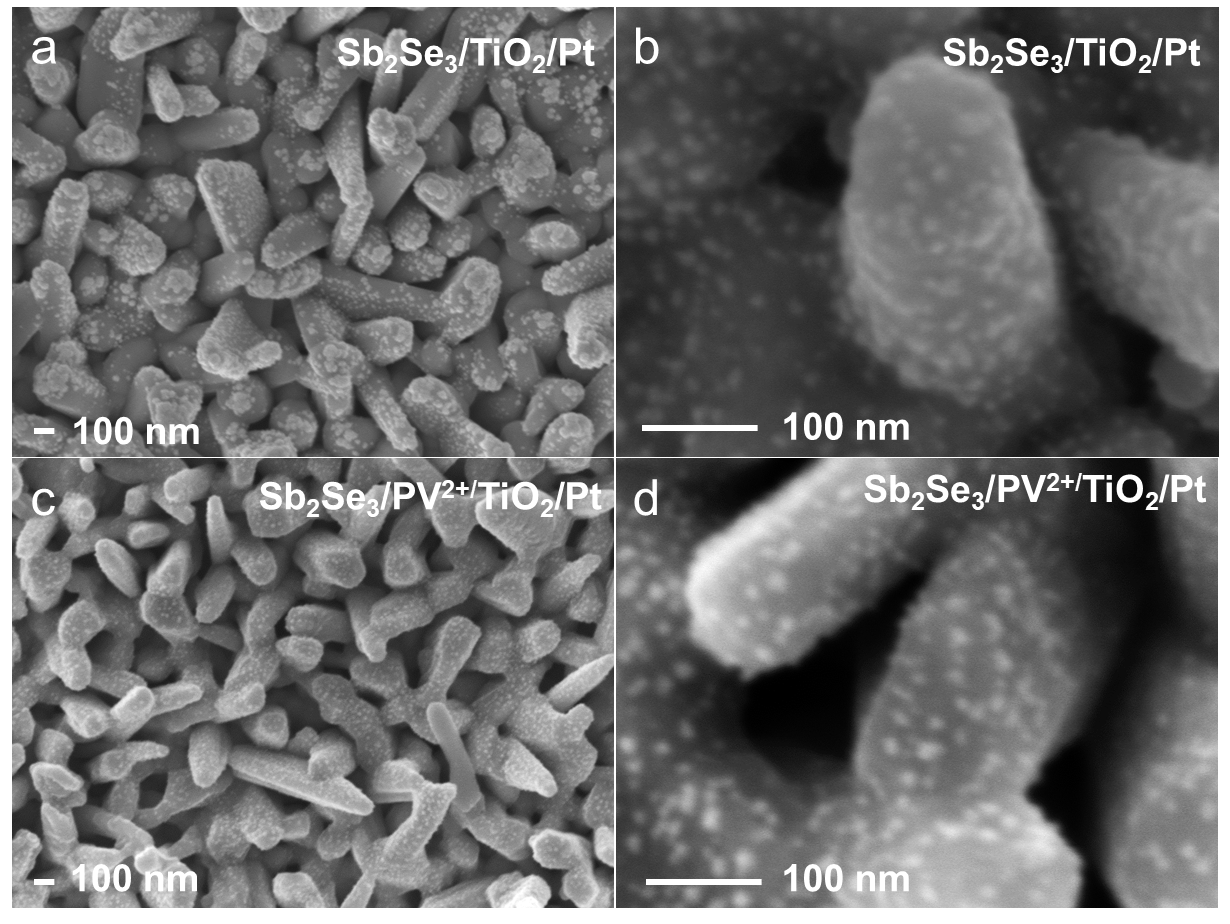


**Fig. S17.** **SEM images of (a) (b) Sb_2_Se_3_/TiO_2_/Pt and (c) (d) Sb_2_Se_3_/PV^2+^/TiO_2_/Pt electrodes.**


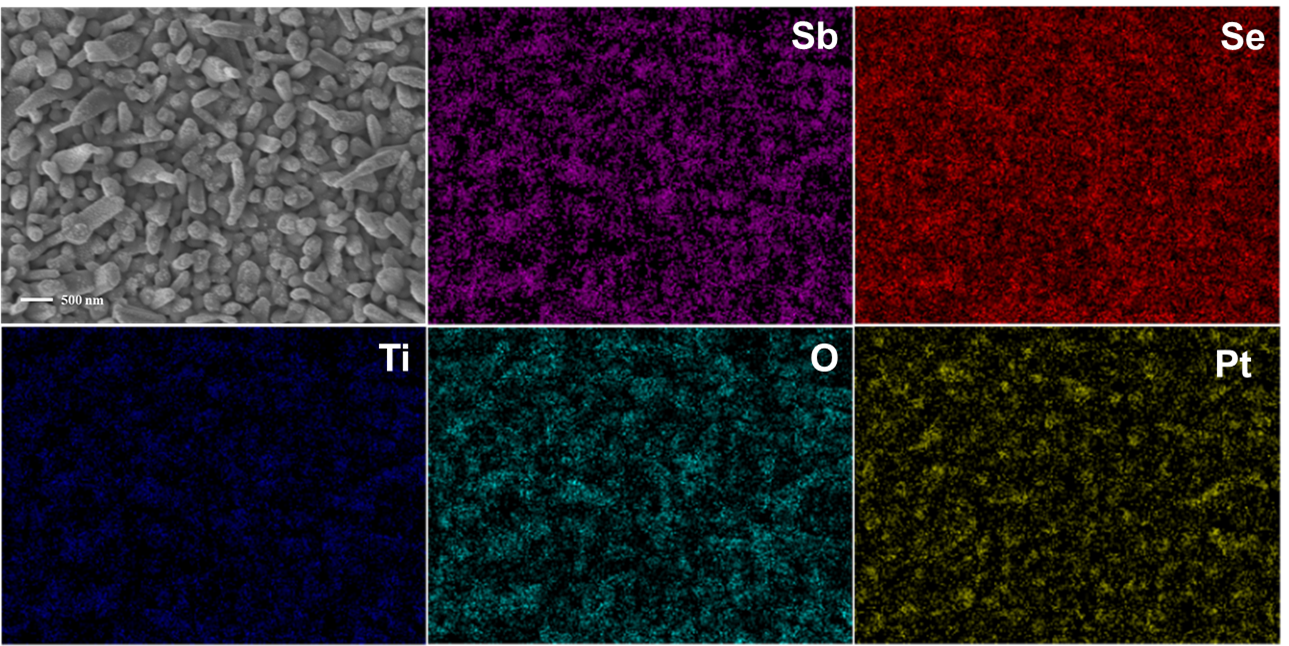


**Fig. S18. SEM image and EDS elemental mapping of the Sb_2_Se_3_/TiO_2_/Pt film. Elements detected: Se, Sb, Ti, O, Pt .**


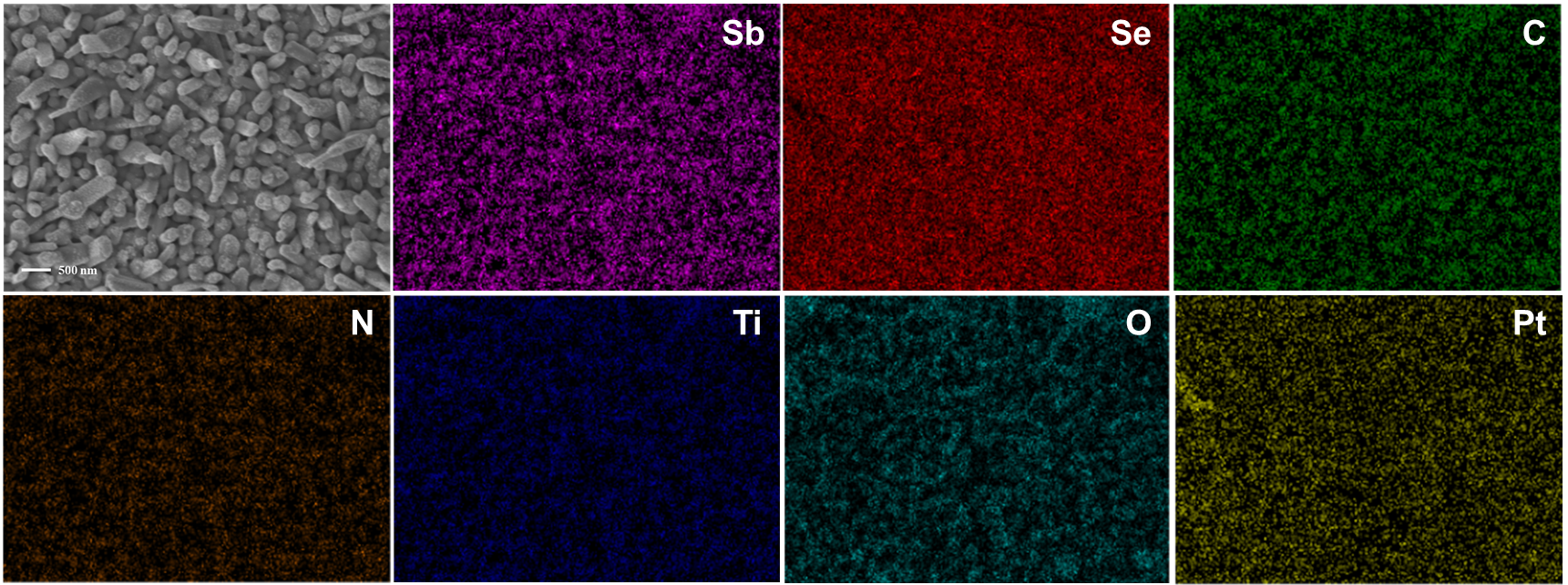


**Fig. S19. SEM image and EDS elemental mapping of the Sb_2_Se_3_/PV^2+^/TiO_2_/Pt film. Elements detected: Se, Sb, C, N, Ti, O, Pt.**


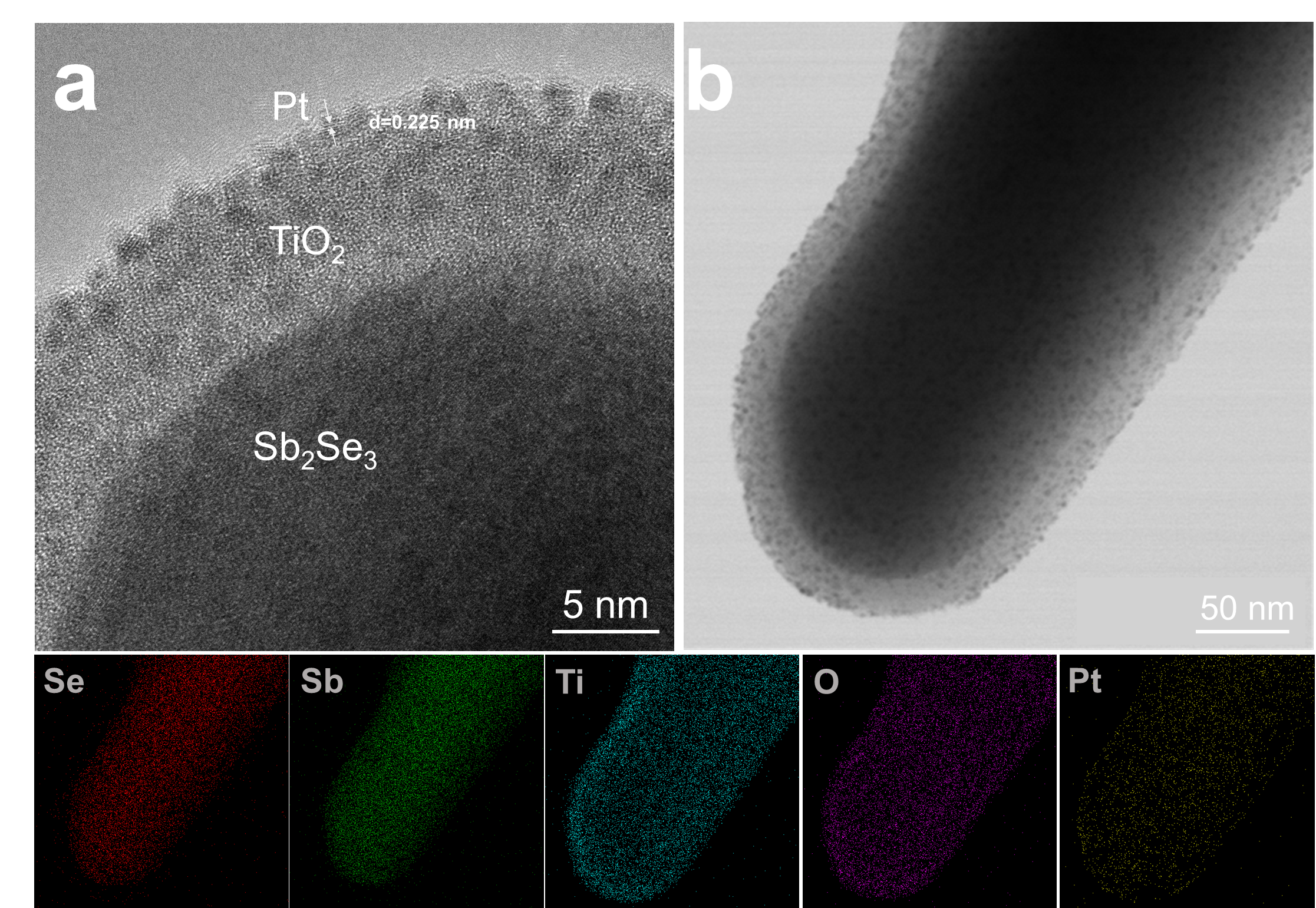


**Fig. S20.** (a) HRTEM image for Sb_2_Se_3_/TiO_2_/Pt. (b) TEM image and EDS elemental mapping of the Sb_2_Se_3_/TiO_2_/Pt film. Elements detected: Se, Sb, Ti, O, Pt;

**Fig. S21.** **LSV curves of Sb_2_Se_3_/Pt and Sb_2_Se_3_/RV^2+^/Pt photocathodes under chopped illumination in the 0.1 M H_2_SO_4_ electrolyte.**

**Fig. S22.** (a) *I-t* curve of the Sb_2_Se_3_/PV^2+^/TiO_2_/Pt photocathode at 0.17 V vs. RHE under illumination (100 mW cm^-2^)

**Fig. S23.**  **Faraday efficiency of Sb_2_Se_3_/PV^2+^/TiO_2_/Pt photocathode for HER.** Hydrogen evolution detected by gas chromatography and the charge passed during the photolysis of Sb_2_Se_3_/RV^2+^/Pt photocathode at an applied potential of 0.17 V vs. RHE

**Fig.S24.** **UV-vis absorbance spectra of Sb_2_Se_3_ and Sb_2_Se_3_/PV^2+^ thin films.**


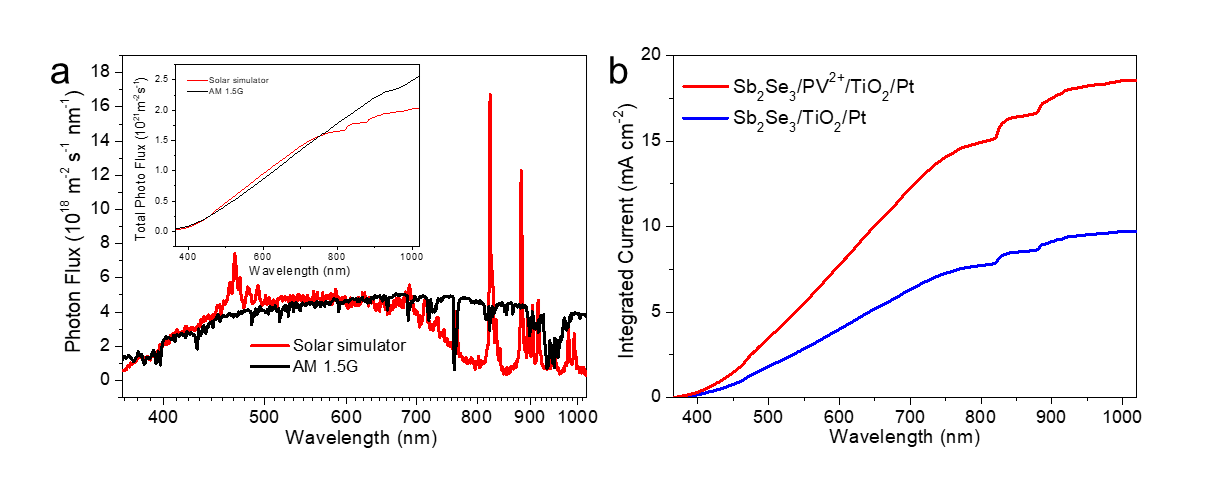


**Fig. S25.** (a) Solar irradiance of the AM 1.5G (ASTM G17303) and the solar simulator. (b) The calculated photocurrents of Sb_2_Se_3_/TiO_2_/Pt and Sb_2_Se_3_/PV^2+^/TiO_2_/Pt at 0 V vs. RHE by integrating IPCE over the light spectrum of the solar simulator.


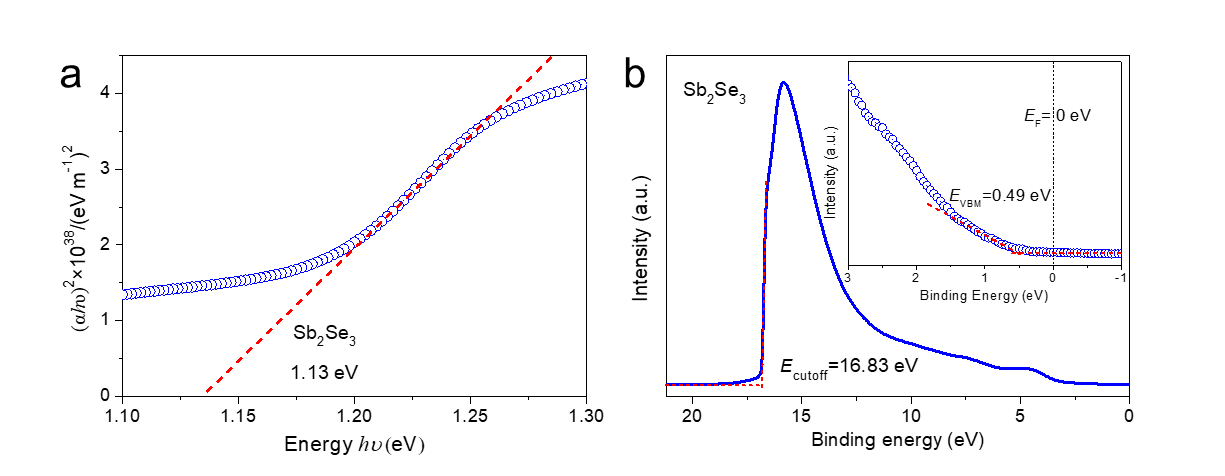


**Fig. S26.** (a) Tauc plot of Sb_2_Se_3_ calculated by the UV-vis absorbance spectrum. The bandgap of Sb_2_Se_3_ is determined to be 1.13 eV. (b) UPS spectra of the Sb_2_Se_3_ for VB determination. The insert shows the enlarged valence band position of Sb_2_Se_3_.

The work function (*E_work_* corresponding to the Fermi level), valence band (VB), and conduction band (CB) of Sb_2_Se_3_ were determined according to the following equations.

|  | $E_{work}=h\nu-\left(E_{cutoff}-E_{F}\right) =21.2-\left(16.83-0\right)=4.37\text{ eV}$ | *Eq. S1* |
| --- | --- | --- |
|  | $E_{VB}=E_{\text{work}}+E_{VBM}=4.37+0.49=4.86\text{ eV}$ | *Eq. S2* |
|  | $E_{CB}=E_{VB}-E_{\text{g}} =4.86-1.13=3.73\text{ eV}$ | *Eq. S3* |

Where, the *E*_cutoff_ is the cut-off energy edge, the *E*_F_ is initial edge energy, the *E*_VBM_ is the energy from valence band maximum to Fermi level, the *E*_g_ is bandgap energy (obtained by UV-vis spectra), the *E*_VB_ is valance band energy and the *E*_CB_ is conduction band energy.

**Fig. S27.** **Mott−Shockley plots at 1 kHz for TiO_2_ photoanodes in 0.1 M H_2_SO_4_.** The flat-band potential (*E*_fb_) is estimated from the intercepts of the extrapolated lines.


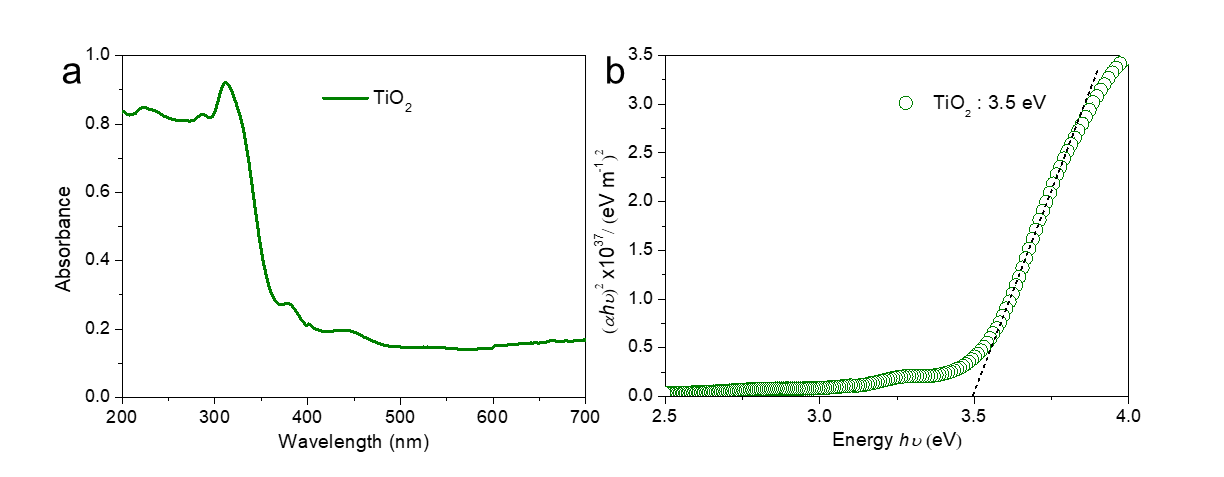


**Fig. S28.** (a) UV-vis absorbance spectrum of TiO_2_. (b) Tauc plot of TiO_2_ calculated by the UV-vis absorbance spectrum.

In order to estimate band positions of TiO_2_, the flat-band potential (*E*_fb_) of TiO_2_ was measured by constructing ESI measurement to drew the Mott−Shockley plots. It was reported that *E*_fb_ is about 100 mV below the CB edge, the CB of TiO_2_ was estimated as

|  | $E_{CB}= E_{fb}+0.1=0.12+0.1=0.22\text{ V }=-\text{4.22 eV}$. | *Eq. S4* |
| --- | --- | --- |

The VB of TiO_2_ was estimated

|  | $E_{VB}= E_{CB}-E_{g}=-4.22-3.5=-7.72\text{ eV }=3.28\text{ V}$. | *Eq. S5* |
| --- | --- | --- |

**Fig. S29.** (a) Cyclic voltammogram (CV) of methylviologen measured in 0.1 M H_2_SO_4_ solution. Inset shows the corresponding differential pulse voltammogram (DPV).

The catalyst of Pt nanoparticles was deposited on FTO glass by electrodepositing at a constant current of -50 μA for 20 min. The LSV curve for Pt/FTO in 0.1 M H_2_SO_4_ was shown in Fig.S30, in which the onset potential of Pt for hydrogen evolution was near to 0 V vs. RHE.

**Fig. S30.** **The LSV curve for Pt/FTO in 0.1 M H_2_SO_4_.** The sweep rate was 10 mV s^-1^.


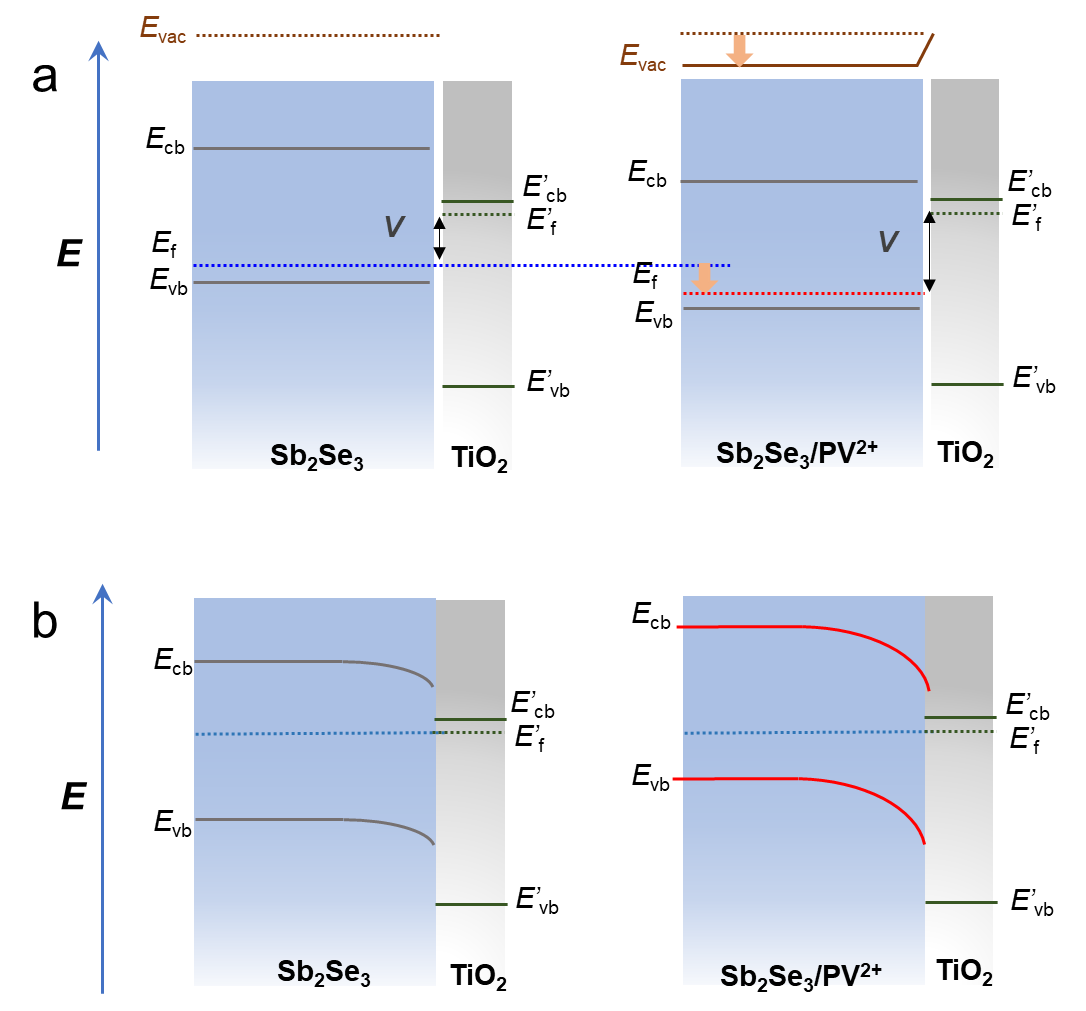


**Fig. S31.**  **Band diagrams of Sb_2_Se_3_ with and without PV^2+^ in flat band condition (a) and in equilibrium (b).** The band bending in the TiO_2_ was omitted.

**Table S1.** **The TRPL fitting data with a bi-exponential fitting model for the Sb_2_Se_3_, Sb_2_Se_3_/TiO_2_ and Sb_2_Se_3_/PV^2+^/TiO_2_**

|  | τ_1_  (ns) | τ_2_  (ns) |
| --- | --- | --- |
| **Sb_2_Se_3_** | 0.39 | 0.22 |
| **Sb_2_Se_3_/TiO_2_** | 0.41 | 0.21 |
| **Sb_2_Se_3_/RV^2+^/ TiO_2_** | 0.39 | 0.12 |

**Fig. S32.** **Normalized plots of the photocurrent density–time dependence, the inset shows *J–V* curves measured at 0 V vs. RHE under chopped light illumination**

**Table S2**. **The values of characteristic lifetimes τ_scr_ and τ_int_ calculated from IMVS curves**

|  | τ_n_  (μs) | τ_int_  (ms) |
| --- | --- | --- |
| **Sb_2_Se_3_/TiO_2_/Pt** | 95.2 | 19.5 |
| **Sb_2_Se_3_/RV^2+^/ TiO_2_/Pt** | 113.9 | 37.9 |

**Table S3.** **The values of resistances and capacitances in fitted EC**

|  | R_S_  (Ω) | R_Bulk_  (Ω) | C_bulk_  (μF) | R_ct_  (Ω) | R_ct_  (Ω) |
| --- | --- | --- | --- | --- | --- |
| **Sb_2_Se_3_/TiO_2_/Pt** | 9.87 | 22.9 | 183 | 13.7 | 7.89 |
| **Sb_2_Se_3_/RV^2+^/ TiO_2_/Pt** | 9.68 | 18.9 | 213 | 5.88 | 11.1 |
